# On the existence of solutions to fractional differential equations involving Caputo *q*-derivative in Banach spaces

**DOI:** 10.1016/j.heliyon.2024.e40876

**Published:** 2024-12-09

**Authors:** Isra Al-Shbeil, Houari Bouzid, Benali Abdelkader, Alina Alp Lupas, Mohammad Esmael Samei, Reem K. Alhefthi

**Affiliations:** a Department of Mathematics, Faculty of Sciences, The University of Jordan, 11942 Amman, Jordan; b Department of Mathematics, Faculty of Exact Science and Informatics, Hassiba Benbouali University of Chlef, Ouled Fares, Chlef 02000, Laboratory of Mathematics and Applications (LMA), Algeria; c Department of Mathematics and Computer Sciences, University of Oradea, 10087 Oradea, Romania; d Department of Mathematics, Faculty of Science, Bu-Ali Sina University, Hamedan, Iran; e Department of Mathematics, College of Science, King Saud University, P.O. Box 2455, Riyadh 11451, Saudi Arabia

**Keywords:** 26A33, 34A08, 34B15, Nonliner fractional equation, Leray-Shauders alternative, Existence, *q*-R-L integral

## Abstract

The generalization of BVPs always covers a wide range of equations. Our choice in this research is the generalization of Caputo-type fractional discrete differential equations that include two or more fractional *q*-integrals. We analyze the existence and uniqueness of solutions to the multi-point nonlinear BVPs base on fixed point theory, including fixed point theorem of Banach, Leray-nonlinear Schauder's alternative, and Leray-degree Schauder's theory. Finally, several examples are presented to demonstrate accuracy of our results.

## 1 Introduction

Fractional derivatives arise in many physical processes, such as charge transport in amorphous semiconductors, electrochemistry, and materials science, and are often modeled using differential equations (DEs) of fractional order [1], [2], [3], [4], [5], [6], [7], [8], [9], [10]. In recent years, there has been a growing interest in fractional differential equations (FDEs), employing various operators like Riemann-Liouville (RL) [11], [12], [13], [14], Caputo [15], [16], [17], [18], [19], Hadamard [20], [21], [22], [23], [24], *q*-fractional [25], [26], [27], and Δ-Hilfer [28], [29].

In 1910, Frank Hilton Jackson introduced and advanced *q*-calculus by defining the *q*-analog of the ordinary derivative [30]. Recognizing the importance of this theory, *q*-differential equations (*q*-DEs) and related operators have been extensively studied, leading to the establishment of the *q*-derivative (a generalized form of the classical derivative), *q*-integral, *q*-factorial, and various specialized functions by numerous researchers [31], [32], [33], [34], [35], [36]. Qarout et al. investigated a class of boundary value problems (BVPs) involving one-dimensional higher-order semi-linear Caputo-type FDEs with nonlocal multi-point discrete and boundary conditions of integral type, using standard tools from fixed point theory (FPT) [37]. Houas and Samei explored the existence, uniqueness, and Hyers-Ulam stability of solutions for the sequential *q*-fractional Duffing-Rayleigh problem in the form 0<q<1:

{Dqζ1C(Dqζ2C(Dqζ3C+θ))ξ(ϑ)=p(ϑ)−ϕ(ϑ,ξ(ϑ))−λ℧(ϑ,ξ(ϑ),Dqγ1Cξ(ϑ))−δψ(ϑ,ξ(ϑ),Dqγ2Cξ(ϑ)),ϑ∈[0,1],ξ(0)=Λ1,(Dqζ3C+θ)ξ(1)=Λ2,Dqζ2C((Dqζ3C+θ))ξ(ω)=Λ3,Λi∈R,i=1,2,3,

where $\theta ,\;\lambda ,\;\delta \in R^{+},\;0<\omega <1$, $0<\zeta_{1},\;\zeta_{2},\;\zeta_{3}<1,\;\gamma_{1}<\zeta_{3},\;\gamma_{2}<\zeta_{3}$ and DqϰC,ϰ∈{ζ1,ζ2,ζ3,γ1,γ2} is the Caputo fractional *q*−derivative of order *ϰ*, p:[0,1]→R,ϕ:[0,1]×R→R and $℧,\psi :\left[0,1\right]\times R^{2}\to R$ are given continuous functions [32]. In 2023, Patle et al. by obtaining best proximity point results, demonstrated the existence of optimum solutions for a system right sided *ψ*-Hilfer FDEs of arbitrary order with initial conditions,

{Da+ζ2,ν;ψHξ(ϑ)=W(ϑ,ξ(ϑ)),Da+ζ2,ν;ψHξ´(ϑ)=W´(ϑ,ξ´(ϑ)),Ia+(1−ν)(1−ζ2);ψξ(a)=ςa,Ia+(1−ν)(1−ζ2);ψξ´(a)=ς´a,

for x∈(a,ϑ], where Da+ν,ζ2;ψH is the left sided *ψ*-Hilfer fractional differential operator of order $0<\zeta_{2}<1$ and type 0<ν≤1, $I_{a^{+}}^{(1-\nu )(1-\zeta_{2});\psi }$ is the RL fractional integral of order $\left(1-\nu )(1-\zeta_{2}\right)$; the state ξ(⋅) takes the values from X, and $W:[a,\vartheta ]\times B_{1}\to X$ and $\overset{´}{W}:[a,\vartheta ]\times B_{2}\to X$, are given mappings [28]. Furthermore, many authors have obtained the existence and uniqueness of solutions for various classes of FDEs by using various nonlinear analysis techniques. As an example, we recommend that the reader review the references listed in [38], [39], [40], [41], [42], [43].

This article analyzes nonlinear BVP of fractional *q*-differential equations (FqDEs) with two RL *q*-integrals of fractional order as follows,

(1.1) {Dqγ1Cξ(ϑ)=Δ(ϑ,ξ(ϑ))+∑ı˙=1mƛı˙Iqγ2ı˙Δˆı˙(ϑ,ξ(ϑ)),0<q<1,ϑ∈[0,T],Iq2−γ1ξ(0)=0,Dq2−γ1Cξ(T)=∑ȷ˙=1lAȷ˙Iqγ1−1ξ(ζ1ȷ˙),0<ζ1ȷ˙<T,

where Dqγ1C is the fractional *q*-derivative of the Caputo type of orders $\gamma_{1}\in (1,2]$, $I_{q}^{\gamma_{2}}$ is the RL fractional integral of order $\gamma_{2}>0$, ${ƛ}_{\overset{˙}{ı}}$, $A_{\overset{˙}{ȷ}}\in R$ and Δ, ${\overset{ˆ}{\Delta }}_{i}\in C([0,T]\times R)$, $1\leq \overset{˙}{ı}\leq m$, $1\leq \overset{˙}{ȷ}\leq l$, l≥2 are continuous functions. Theorems such as Banach's FPT, Leray-Schauder's nonlinear alternative and degree theory have been used to investigate the existence of the solution for BVP (1.1).

## 2 Preliminaries

Let 0<q≠1 and consider a *q*-real number ${\left[a\right]}_{q}=\frac{1-q^{a}}{1-q}$, for a∈R. The *q*-analogue of the Pochhammer symbol (*q*-shifted factorial) is defined as

$${\left(a;q\right)}_{k}=\left\{ \begin{array}{cc} 1,\; & k=0,\\ \prod_{\overset{˙}{ȷ}=0}^{k-1}(1-aq^{\overset{˙}{ȷ}}),\; & k\in N. \end{array}\right.$$

The *q*-analogue of the exponent ${\left(a-\overset{´}{a}\right)}^{k}$ is expressed by, for $a,\overset{´}{a}\in R$,

$${\left(a-\overset{´}{a}\right)}_{k}=\left\{ \begin{array}{cc} 1,\; & k=0,\\ \prod_{\overset{˙}{ȷ}=0}^{k-1}(a-\overset{´}{a}q^{\overset{˙}{ȷ}}),\; & k\in N. \end{array}\right.$$

And, the *q*-factorial by

$${\left[k\right]}_{q}=\prod_{\overset{˙}{ȷ}=0}^{k-1}{\left[r\right]}_{q}=\frac{{\left(q;q\right)}_{k}}{{\left(1-q\right)}^{k}},\;k\in N.$$

Definition 2.1 **[44]**

The *q*-gamma function $\Gamma_{q}(\gamma_{1})$ is defined as

$$\Gamma_{q}(\gamma_{1})=\frac{{\left(1-q\right)}^{\left(\gamma_{1}-1\right)}}{{\left(1-q\right)}^{\gamma_{1}-1}}={\left(q;q\right)}_{\gamma_{1}-1}{\left(1-q\right)}^{\gamma_{1}-1},\;\gamma_{1}\in C\setminus \{-n\;:\;n\in N\}\cup \{0\},$$

with $\Gamma_{q}(\gamma_{1}+1)={\left[\gamma_{1}\right]}_{q}\Gamma_{q}(\gamma_{1})$.

Definition 2.2 **[45]**

For the given function *ξ* which is defined on [0,1], the RL *q*-integral of fractional order $\gamma_{1}\geq 0$ is $\left(I_{q}^{0}\xi )(\vartheta )=\xi (\vartheta \right)$ and

$$I_{q}^{\gamma_{1}}\xi \left(\vartheta \right)=\int_{0}^{\vartheta }\frac{{\left(\vartheta -qs\right)}^{\left(\gamma_{1}-1\right)}}{\Gamma_{q}(\gamma_{1})}\xi (s)\;d_{q}s=\vartheta^{\gamma_{1}}{\left(1-q\right)}^{\gamma_{1}}\overset{\infty }{\sum_{k=0}}q^{k}\frac{{\left(q^{\gamma_{1}};q\right)}_{k}}{{\left(q;q\right)}_{k}}\xi (\vartheta q^{k}),$$

for $\gamma_{1}>0$, ϑ∈[0,1].

Definition 2.3 **[44]**

The Caputo fractional *q*-derivative of order $\gamma_{1}(n-1,n)$ of the continuous functions ξ:[0,T]→R in the sens of Caputo, denoted by Dqγ1C is defined by

(Dqγ1Cξ)(ϑ)=Iq[γ1]−γ1Dq[γ1]ξ(ϑ),

where $\left[\gamma_{1}\right]$ is the smallest integer greater than or equal to $\gamma_{1}$.

Next, we will remember some properties of fractional R-L *q*-integral and Caputo *q*-derivative [44, Theorem 5.2].

Lemma 2.4 **[44]**

*Let*$\gamma_{1}>0$*and*k∈N*. Then,*

Iqγ1CDqγ1ξ(ϑ)=ξ(ϑ)−∑k=0[γ1]−1ckϑk,Dqγ1CIqγ1ξ(ϑ)=ξ(ϑ),

*for each*ϑ∈[0,T]*, where*$k=1,\ldots ,[\gamma_{1}]-1$*and*$[\gamma_{1}]=k-1$*.*

## 3 Main results

Consider F:=C([0,T],R) with the norm $\left\|\xi \right\|=\sup_{\vartheta \in [0,T]}\left|\xi (\vartheta )\right|$. To solve problem (1.1), we need to use the following important idea.

Lemma 3.1 *Let*Θ∈F*and*0<q<1*. Then, the unique solution of the* BVP*,*

(3.1) {Dqγ1Cξ(ϑ)=Θ(ϑ),ϑ∈[0,T],Iq2−γ1ξ(0)=0,Dq2−γ1Cξ(T)=∑ȷ˙=1lAȷ˙Iqγ1−1ξ(ζ1ȷ˙),0<ζ1ȷ˙<T,

*is given by*

$$\xi (\vartheta )=\int_{0}^{\vartheta }\frac{{\left(\vartheta -qs\right)}^{\left(\gamma_{1}-1\right)}}{\Gamma_{q}(\gamma_{1})}\Theta (s)\;d_{q}s+\frac{\Gamma_{q}(\gamma_{1}+1)\vartheta }{{\left[\gamma_{1}\right]}_{q}T^{\gamma_{1}-1}-E}[\frac{1}{\Gamma_{q}(2\gamma_{1}-1)}\overset{l}{\sum_{\overset{˙}{ȷ}=1}}A_{\overset{˙}{ȷ}}\;\;\int_{0}^{{\zeta_{1}}_{\overset{˙}{ȷ}}}{\left({\zeta_{1}}_{\overset{˙}{ȷ}}-qs\right)}^{\left(2\gamma_{1}-2\right)}\Theta (s)\;d_{q}s-\int_{0}^{T}\frac{{\left(T-qs\right)}^{\left(2\gamma_{1}-3\right)}}{\Gamma_{q}(2\gamma_{1}-2)}\Theta (s)\;d_{q}s],$$

*where* ${\left[\gamma_{1}\right]}_{q}T^{\gamma_{1}-1}\neq E=\overset{l}{\sum_{\overset{˙}{ȷ}=1}}A_{\overset{˙}{ȷ}}{\zeta_{1}}_{\overset{˙}{ȷ}}^{\gamma_{1}}$*.*

Proof Applying $I_{q}^{\gamma_{1}}$ on FqDE in (3.1), we get

$$\xi (\vartheta )=\int_{0}^{\vartheta }\frac{{\left(\vartheta -qs\right)}^{\left(\gamma_{1}-1\right)}}{\Gamma_{q}(\gamma_{1})}\Theta (s)\;d_{q}s+c_{0}+c_{1}\vartheta ,$$

for some constants $c_{0};c_{1}\in R$. Since $I_{q}^{2-\gamma_{1}}\xi (0)=0$, we have $c_{0}=0$. Besides,

Dq2−γ1Cξ(ϑ)=Iq2γ1−2Θ(ϑ)+c1ϑγ1−1Γq(γ1),Iqγ1−1ξ(ϑ)=Iq2γ1−1Θ(ϑ)+c1ϑγ1Γq(γ1+1).

From Dq2−γ1Cξ(T)=∑ȷ˙=1lAȷ˙Iqγ1−1ξ(ζ1ȷ˙), we have

(3.2) $$c_{1}=\frac{\Gamma_{q}(\gamma_{1}+1)}{{\left[\gamma_{1}\right]}_{q}T^{\gamma_{1}-1}-E}[\frac{1}{\Gamma_{q}(2\gamma_{1}-1)}\overset{l}{\sum_{\overset{˙}{ȷ}=1}}A_{\overset{˙}{ȷ}}\int_{0}^{{\zeta_{1}}_{\overset{˙}{ȷ}}}{\left({\zeta_{1}}_{\overset{˙}{ȷ}}-qs\right)}^{\left(2\gamma_{1}-2\right)}\Theta (s)\;d_{q}s\;\;-\int_{0}^{T}\frac{{\left(T-qs\right)}^{\left(2\gamma_{1}-3\right)}}{\Gamma_{q}(2\gamma_{1}-2)}\Theta (s)\;d_{q}s],$$

where $E=\sum_{j=1}^{k}B_{j}{\zeta_{1}}_{j}^{\gamma_{1}}$ and ${\left[\gamma_{1}\right]}_{q}T^{\gamma_{1}-1}\neq E$. Thus,

(3.3) $$\xi (\vartheta )=\int_{0}^{\vartheta }\frac{{\left(\vartheta -qs\right)}^{\left(\gamma_{1}-1\right)}}{\Gamma_{q}(\gamma_{1})}\Theta (s)\;d_{q}s+\frac{\Gamma_{q}(\gamma_{1}+1)\vartheta }{{\left[\gamma_{1}\right]}_{q}T^{\gamma_{1}-1}-E}[\frac{1}{\Gamma_{q}(2\gamma_{1}-1)}\overset{l}{\sum_{\overset{˙}{ȷ}=1}}A_{\overset{˙}{ȷ}}\;\;\int_{0}^{{\zeta_{1}}_{\overset{˙}{ȷ}}}{\left({\zeta_{1}}_{\overset{˙}{ȷ}}-qs\right)}^{\left(2\gamma_{1}-2\right)}\Theta (s)\;d_{q}s-\int_{0}^{T}\frac{{\left(T-qs\right)}^{\left(2\gamma_{1}-3\right)}}{\Gamma_{q}(2\gamma_{1}-2)}\Theta (s)\;d_{q}s].$$

The proof is complete. □

Based on Lemma 3.1, we create a new operator Θ:F→F by:

(3.4) $$\Theta \xi (\vartheta )=\int_{0}^{\vartheta }\frac{{\left(\vartheta -qs\right)}^{\left(\gamma_{1}-1\right)}}{\Gamma_{q}(\gamma_{1})}\Delta (s,\xi (s))\;d_{q}s+\overset{m}{\sum_{\overset{˙}{ı}=1}}{ƛ}_{\overset{˙}{ı}}\int_{0}^{\vartheta }\frac{{\left(\vartheta -qs\right)}^{\left({\gamma_{2}}_{\overset{˙}{ı}}+\gamma_{1}-1\right)}}{\Gamma_{q}({\gamma_{2}}_{\overset{˙}{ı}}+\gamma_{1})}{\overset{ˆ}{\Delta }}_{\overset{˙}{ı}}(s,\xi (s))\;d_{q}s\;\;+\frac{\Gamma_{q}(\gamma_{1}+1)\vartheta }{{\left[\gamma_{1}\right]}_{q}T^{\gamma_{1}-1}-E}[\overset{l}{\sum_{\overset{˙}{ȷ}=1}}A_{\overset{˙}{ȷ}}\int_{0}^{{\zeta_{1}}_{\overset{˙}{ȷ}}}\frac{{\left({\zeta_{1}}_{\overset{˙}{ȷ}}-qs\right)}^{\left(2\gamma_{1}-2\right)}}{\Gamma_{q}(2\gamma_{1}-1)}\Delta (s,\xi (s))\;d_{q}s\;\;+\overset{m}{\sum_{\overset{˙}{ı}=1}}\overset{l}{\sum_{\overset{˙}{ȷ}=1}}A_{\overset{˙}{ȷ}}{ƛ}_{\overset{˙}{ı}}\int_{0}^{{\zeta_{1}}_{\overset{˙}{ȷ}}}\frac{{\left({\zeta_{1}}_{\overset{˙}{ȷ}}-qs\right)}^{\left(2\gamma_{1}+{\gamma_{2}}_{\overset{˙}{ı}}-2\right)}}{\Gamma_{q}(2\gamma_{1}+{\gamma_{2}}_{\overset{˙}{ı}}-1)}{\overset{ˆ}{\Delta }}_{\overset{˙}{ı}}(s,\xi (s))\;d_{q}s\;\;-\int_{0}^{T}\frac{{\left(T-qs\right)}^{\left(2\gamma_{1}-3\right)}}{\Gamma_{q}(2\gamma_{1}-2)}\Delta (s,\xi (s))\;d_{q}s-\overset{m}{\sum_{\overset{˙}{ı}=1}}{ƛ}_{\overset{˙}{ı}}\int_{0}^{T}\frac{{\left(T-qs\right)}^{\left(2\gamma_{1}+{\gamma_{2}}_{\overset{˙}{ı}}-3\right)}}{\Gamma_{q}(2\gamma_{1}+{\gamma_{2}}_{\overset{˙}{ı}}-2)}{\overset{ˆ}{\Delta }}_{\overset{˙}{ı}}(s,\xi (s))\;d_{q}s].$$

We know, finding a FP of the operator *Θ* is the same as the solution to BVP (1.1). Observe that the existence of a FP for the operator *Θ* implies the existence of a solution for the multi-point BVP (1.1). We point out the expression ℵ as,

(3.5) $$ℵ=T^{\gamma_{1}}{\left(1-q\right)}^{\gamma_{1}}\overset{\infty }{\sum_{l=0}}q^{l}\frac{{\left(q^{\gamma_{1}};q\right)}_{l}}{{\left(q;q\right)}_{l}}+\overset{m}{\sum_{\overset{˙}{ı}=1}}|{ƛ}_{\overset{˙}{ı}}|T^{{\gamma_{2}}_{\overset{˙}{ı}}+\gamma_{1}}{\left(1-q\right)}^{{\gamma_{2}}_{\overset{˙}{ı}}+\gamma_{1}}\overset{\infty }{\sum_{l=0}}q^{l}\frac{{\left(q^{{\gamma_{2}}_{\overset{˙}{ı}}+\gamma_{1}};q\right)}_{l}}{{\left(q;q\right)}_{l}}\;\;+\frac{\Gamma_{q}(\gamma_{1}+1)T}{\left|{\left[\gamma_{1}\right]}_{q}T^{\gamma_{1}-1}-E\right|}[\overset{l}{\sum_{\overset{˙}{ȷ}=1}}|A_{\overset{˙}{ȷ}}|{\zeta_{1}}_{\overset{˙}{ȷ}}^{2\gamma_{1}-1}{\left(1-q\right)}^{2\gamma_{1}-1}\overset{\infty }{\sum_{l=0}}q^{l}\frac{{\left(q^{2\gamma_{1}-1};q\right)}_{l}}{{\left(q;q\right)}_{l}}\;\;+\overset{m}{\sum_{\overset{˙}{ı}=1}}\overset{l}{\sum_{\overset{˙}{ȷ}=1}}\;|{ƛ}_{\overset{˙}{ı}}|\;|A_{\overset{˙}{ȷ}}|\;{\zeta_{1}}_{\overset{˙}{ȷ}}^{2\gamma_{1}+{\gamma_{2}}_{\overset{˙}{ı}}-1}{\left(1-q\right)}^{2\gamma_{1}+{\gamma_{2}}_{\overset{˙}{ı}}-1}\overset{\infty }{\sum_{l=0}}q^{l}\frac{{\left(q^{2\gamma_{1}+{\gamma_{2}}_{\overset{˙}{ı}}-1};q\right)}_{l}}{{\left(q;q\right)}_{l}}\;\;+T^{2\gamma_{1}-2}{\left(1-q\right)}^{2\gamma_{1}-2}\overset{\infty }{\sum_{l=0}}q^{l}\frac{{\left(q^{2\gamma_{1}-2};q\right)}_{l}}{{\left(q;q\right)}_{l}}\;\;+\overset{m}{\sum_{\overset{˙}{ı}=1}}|{ƛ}_{\overset{˙}{ı}}|T^{2\gamma_{1}+{\gamma_{2}}_{\overset{˙}{ı}}-2}{\left(1-q\right)}^{2\gamma_{1}+{\gamma_{2}}_{\overset{˙}{ı}}-2}\overset{\infty }{\sum_{l=0}}q^{l}\frac{{\left(q^{2\gamma_{1}+{\gamma_{2}}_{\overset{˙}{ı}}-2};q\right)}_{l}}{{\left(q;q\right)}_{l}}].$$

In the sequel, we investigate existence and uniqueness results for multi-point BVP (1.1) base on a variety of FPTs. First, we employing Banach's FPT.

Theorem 3.2 *Take* Δ*,* ${\overset{ˆ}{\Delta }}_{\overset{˙}{ı}}\in C([0,T]\times R)$*,* $\overset{˙}{ı}=1,\ldots ,m$ *where*

(H1) *there exist*$\psi_{\overset{˙}{ı}}\geq 0$*,*$\overset{˙}{ı}=1,\ldots ,m+1$*, s.t.*∀ϑ∈[0,T]*and ξ,*$\overset{ˆ}{\xi }\in R$*, we have*
  $$|\Delta (\vartheta ,\xi )-\Delta (\vartheta ,\overset{ˆ}{\xi })|\leq \psi_{1}^{⁎}|\xi -\overset{ˆ}{\xi }|,\;|{\overset{ˆ}{\Delta }}_{\overset{˙}{ı}}(\vartheta ,\xi )-{\overset{ˆ}{\Delta }}_{\overset{˙}{ı}}(\vartheta ,\overset{ˆ}{\xi })|\leq \psi_{\overset{˙}{ı}+1}^{⁎}|\xi -\overset{ˆ}{\xi }|,$$
  *for*$\overset{˙}{ı}=1,2,\ldots ,l$*.*

*Then the multi-point* BVP (1.1) *has a unique solution provided by* ${\overset{˘}{\psi }}^{⁎}ℵ<1$*, where* $\zeta_{2}=\max\{\psi_{\overset{˙}{ı}}^{⁎}\;:\;\overset{˙}{ı}=1,2,\ldots ,m+1\}$*,* ℵ *given by* (3.5)*.*

Proof Let us define $L=\max\{L_{\overset{˙}{ı}}\;:\;\overset{˙}{ı}=1,2,\ldots ,m+1\}$, where

$$L_{1}=\underset{\vartheta \in [0,T]}{\sup}|\Delta \left(\vartheta ,0\right)|,\;L_{\overset{˙}{ı}+1}=\underset{\vartheta \in [0,T]}{\sup}|{\overset{ˆ}{\Delta }}_{\overset{˙}{ı}}\left(\vartheta ,0\right)|.$$

Take $r\geq (\zeta_{2}r+L)ℵ$, we show that $\Theta {ℷ}_{r}\subset {ℷ}_{r}$, where ${ℷ}_{r}=\{\xi \in F:\left\|\xi \right\|\leq r\}$. For $\xi \in {ℷ}_{r}$ and each ϑ∈[0,T], from the definition of *Θ* and hypothesis (H1), we obtain

$$\left\|\Theta \xi \right\|\leq \underset{\vartheta \in [0,T]}{\sup}\{\int_{0}^{\vartheta }\frac{{\left(\vartheta -qs\right)}^{\left(\gamma_{1}-1\right)}}{\Gamma_{q}(\gamma_{1})}|\Delta (s,\xi (s))|\;d_{q}s\;\;+\overset{m}{\sum_{\overset{˙}{ı}=1}}|{ƛ}_{\overset{˙}{ı}}|\int_{0}^{\vartheta }\frac{{\left(\vartheta -qs\right)}^{\left({\gamma_{2}}_{\overset{˙}{ı}}+\gamma_{1}-1\right)}}{\Gamma_{q}({\gamma_{2}}_{\overset{˙}{ı}}+\gamma_{1})}|{\overset{ˆ}{\Delta }}_{\overset{˙}{ı}}(s,\xi (s))|\;d_{q}s\;\;+\frac{\Gamma_{q}(\gamma_{1}+1)\vartheta }{\left|{\left[\gamma_{1}\right]}_{q}T^{\gamma_{1}-1}-E\right|}(\overset{l}{\sum_{\overset{˙}{ȷ}=1}}|A_{\overset{˙}{ȷ}}|\int_{0}^{{\zeta_{1}}_{\overset{˙}{ȷ}}}\frac{{\left({\zeta_{1}}_{\overset{˙}{ȷ}}-qs\right)}^{\left(2\gamma_{1}-2\right)}}{\Gamma_{q}(2\gamma_{1}-1)}|\Delta (s,\xi (s))|\;d_{q}s\;\;+\overset{m}{\sum_{\overset{˙}{ı}=1}}\overset{l}{\sum_{\overset{˙}{ȷ}=1}}\;|{ƛ}_{\overset{˙}{ı}}|\;|A_{\overset{˙}{ȷ}}|\;\int_{0}^{{\zeta_{1}}_{\overset{˙}{ȷ}}}\frac{{\left({\zeta_{1}}_{\overset{˙}{ȷ}}-qs\right)}^{\left(2\gamma_{1}+{\gamma_{2}}_{\overset{˙}{ı}}-2\right)}}{\Gamma_{q}(2\gamma_{1}+{\gamma_{2}}_{\overset{˙}{ı}}-1)}|{\overset{ˆ}{\Delta }}_{\overset{˙}{ı}}(s,\xi (s))|\;d_{q}s\;\;+\int_{0}^{T}\frac{{\left(T-qs\right)}^{\left(2\gamma_{1}-3\right)}}{\Gamma_{q}(2\gamma_{1}-2)}|\Delta (s,\xi (s))|\;d_{q}s\;\;+\overset{m}{\sum_{\overset{˙}{ı}=1}}|{ƛ}_{\overset{˙}{ı}}|\int_{0}^{T}\frac{{\left(T-qs\right)}^{\left(2\gamma_{1}+{\gamma_{2}}_{\overset{˙}{ı}}-3\right)}}{\Gamma_{q}(2\gamma_{1}+{\gamma_{2}}_{\overset{˙}{ı}}-2)}|{\overset{ˆ}{\Delta }}_{\overset{˙}{ı}}(s,\xi (s))|\;d_{q}s)\}\leq \underset{\vartheta \in [0,T]}{\sup}\{\int_{0}^{\vartheta }\frac{{\left(\vartheta -qs\right)}^{\left(\gamma_{1}-1\right)}}{\Gamma_{q}(\gamma_{1})}(|\Delta (s,\xi (s))-\Delta (s,0)|+|\Delta (s,0)|)\;d_{q}s\;\;+\overset{m}{\sum_{\overset{˙}{ı}=1}}|{ƛ}_{\overset{˙}{ı}}|\int_{0}^{\vartheta }\frac{{\left(\vartheta -qs\right)}^{\left({\gamma_{2}}_{\overset{˙}{ı}}+\gamma_{1}-1\right)}}{\Gamma_{q}({\gamma_{2}}_{\overset{˙}{ı}}+\gamma_{1})}(|{\overset{ˆ}{\Delta }}_{\overset{˙}{ı}}(s,\xi (s))-{\overset{ˆ}{\Delta }}_{\overset{˙}{ı}}(s,0)|+|{\overset{ˆ}{\Delta }}_{\overset{˙}{ı}}(s,0)|)\;d_{q}s\;\;+\frac{\Gamma_{q}(\gamma_{1}+1)\vartheta }{\left|{\left[\gamma_{1}\right]}_{q}T^{\gamma_{1}-1}-E\right|}(\overset{l}{\sum_{\overset{˙}{ȷ}=1}}|A_{\overset{˙}{ȷ}}|\int_{0}^{{\zeta_{1}}_{\overset{˙}{ȷ}}}\frac{{\left({\zeta_{1}}_{\overset{˙}{ȷ}}-qs\right)}^{\left(2\gamma_{1}-2\right)}}{\Gamma_{q}(2\gamma_{1}-1)}(|\Delta (s,\xi (s))-\Delta (s,0)|+|\Delta (s,0)|)\;d_{q}s\;\;+\overset{m}{\sum_{\overset{˙}{ı}=1}}\overset{l}{\sum_{\overset{˙}{ȷ}=1}}|A_{\overset{˙}{ȷ}}||{ƛ}_{\overset{˙}{ı}}|\int_{0}^{{\zeta_{1}}_{\overset{˙}{ȷ}}}\frac{{\left({\zeta_{1}}_{\overset{˙}{ȷ}}-qs\right)}^{\left(2\gamma_{1}+{\gamma_{2}}_{\overset{˙}{ı}}-2\right)}}{\Gamma_{q}(2\gamma_{1}+{\gamma_{2}}_{\overset{˙}{ı}}-1)}(|{\overset{ˆ}{\Delta }}_{\overset{˙}{ı}}(s,\xi (s))-{\overset{ˆ}{\Delta }}_{\overset{˙}{ı}}(s,0)|+|{\overset{ˆ}{\Delta }}_{\overset{˙}{ı}}(s,0)|)\;d_{q}s\;\;+\int_{0}^{T}\frac{{\left(T-qs\right)}^{\left(2\gamma_{1}-3\right)}}{\Gamma_{q}(2\gamma_{1}-2)}(|\Delta (s,\xi (s))-\Delta (s,0)|+|\Delta (s,0)|)\;d_{q}s\;\;+\overset{m}{\sum_{\overset{˙}{ı}=1}}|{ƛ}_{\overset{˙}{ı}}|\int_{0}^{T}\frac{{\left(T-qs\right)}^{\left(2\gamma_{1}+{\gamma_{2}}_{\overset{˙}{ı}}-3\right)}}{\Gamma_{q}(2\gamma_{1}+{\gamma_{2}}_{\overset{˙}{ı}}-2)}(|{\overset{ˆ}{\Delta }}_{\overset{˙}{ı}}(s,\xi (s))-{\overset{ˆ}{\Delta }}_{\overset{˙}{ı}}(s,0)|+|{\overset{ˆ}{\Delta }}_{\overset{˙}{ı}}(s,0)|)\;d_{q}s)\}\leq (\zeta_{2}r+L)\underset{\vartheta \in [0,T]}{\sup}\{\int_{0}^{\vartheta }\frac{{\left(\vartheta -qs\right)}^{\left(\gamma_{1}-1\right)}}{\Gamma_{q}(\gamma_{1})}\;d_{q}s+\overset{m}{\sum_{\overset{˙}{ı}=1}}|{ƛ}_{\overset{˙}{ı}}|\int_{0}^{\vartheta }\frac{{\left(\vartheta -qs\right)}^{\left({\gamma_{2}}_{\overset{˙}{ı}}+\gamma_{1}-1\right)}}{\Gamma_{q}({\gamma_{2}}_{\overset{˙}{ı}}+\gamma_{1})}\;d_{q}s\;\;+\frac{\Gamma_{q}(\gamma_{1}+1)\vartheta }{\left|{\left[\gamma_{1}\right]}_{q}T^{\gamma_{1}-1}-E\right|}(\overset{l}{\sum_{\overset{˙}{ȷ}=1}}|A_{\overset{˙}{ȷ}}|\int_{0}^{{\zeta_{1}}_{\overset{˙}{ȷ}}}\frac{{\left({\zeta_{1}}_{\overset{˙}{ȷ}}-qs\right)}^{\left(2\gamma_{1}-2\right)}}{\Gamma_{q}(2\gamma_{1}-1)}\;d_{q}s\;\;+\overset{m}{\sum_{\overset{˙}{ı}=1}}\overset{l}{\sum_{\overset{˙}{ȷ}=1}}|A_{\overset{˙}{ȷ}}||{ƛ}_{\overset{˙}{ı}}|\int_{0}^{{\zeta_{1}}_{\overset{˙}{ȷ}}}\frac{{\left({\zeta_{1}}_{\overset{˙}{ȷ}}-qs\right)}^{\left(2\gamma_{1}+{\gamma_{2}}_{\overset{˙}{ı}}-2\right)}}{\Gamma_{q}(2\gamma_{1}+{\gamma_{2}}_{\overset{˙}{ı}}-1)}\;d_{q}s\;\;-\int_{0}^{T}\frac{{\left(T-qs\right)}^{\left(2\gamma_{1}-3\right)}}{\Gamma_{q}(2\gamma_{1}-2)}\;d_{q}s+\overset{m}{\sum_{\overset{˙}{ı}=1}}|{ƛ}_{\overset{˙}{ı}}|\int_{0}^{T}\frac{{\left(T-qs\right)}^{\left(2\gamma_{1}+{\gamma_{2}}_{\overset{˙}{ı}}-3\right)}}{\Gamma_{q}(2\gamma_{1}+{\gamma_{2}}_{\overset{˙}{ı}}-2)}\;d_{q}s)\}\leq (\omega r+L)[T^{\gamma_{1}}{\left(1-q\right)}^{\gamma_{1}}\overset{\infty }{\sum_{l=0}}q^{l}\frac{{\left(q^{\gamma_{1}};q\right)}_{l}}{{\left(q;q\right)}_{l}}\;\;+\overset{m}{\sum_{\overset{˙}{ı}=1}}|{ƛ}_{\overset{˙}{ı}}|T^{{\gamma_{2}}_{\overset{˙}{ı}}+\gamma_{1}}{\left(1-q\right)}^{{\gamma_{2}}_{\overset{˙}{ı}}+\gamma_{1}}\overset{\infty }{\sum_{l=0}}q^{l}\frac{{\left(q^{{\gamma_{2}}_{\overset{˙}{ı}}+\gamma_{1}};q\right)}_{l}}{{\left(q;q\right)}_{l}}\;\;+\frac{\Gamma_{q}(\gamma_{1}+1)T}{\left|{\left[\gamma_{1}\right]}_{q}T^{\gamma_{1}-1}-E\right|}(\overset{l}{\sum_{\overset{˙}{ȷ}=1}}|A_{\overset{˙}{ȷ}}|{\zeta_{1}}_{\overset{˙}{ȷ}}^{2\gamma_{1}-1}{\left(1-q\right)}^{2\gamma_{1}-1}\overset{\infty }{\sum_{l=0}}q^{l}\frac{{\left(q^{2\gamma_{1}-1};q\right)}_{l}}{{\left(q;q\right)}_{l}}\;\;+\overset{m}{\sum_{\overset{˙}{ı}=1}}\overset{l}{\sum_{\overset{˙}{ȷ}=1}}|A_{\overset{˙}{ȷ}}||{ƛ}_{\overset{˙}{ı}}|{\zeta_{1}}_{\overset{˙}{ȷ}}^{2\gamma_{1}+{\gamma_{2}}_{\overset{˙}{ı}}-1}{\left(1-q\right)}^{2\gamma_{1}+{\gamma_{2}}_{\overset{˙}{ı}}-1}\overset{\infty }{\sum_{l=0}}q^{l}\frac{{\left(q^{2\gamma_{1}+{\gamma_{2}}_{\overset{˙}{ı}}-1};q\right)}_{l}}{{\left(q;q\right)}_{l}}\;\;+T^{2\gamma_{1}-2}{\left(1-q\right)}^{2\gamma_{1}-2}\overset{\infty }{\sum_{l=0}}q^{l}\frac{{\left(q^{2\gamma_{1}-2};q\right)}_{l}}{{\left(q;q\right)}_{l}}\;\;+\overset{m}{\sum_{\overset{˙}{ı}=1}}|{ƛ}_{\overset{˙}{ı}}|T^{2\gamma_{1}+{\gamma_{2}}_{\overset{˙}{ı}}-2}{\left(1-q\right)}^{2\gamma_{1}+{\gamma_{2}}_{\overset{˙}{ı}}-2}\overset{\infty }{\sum_{l=0}}q^{l}\frac{{\left(q^{2\gamma_{1}+{\gamma_{2}}_{\overset{˙}{ı}}-2};q\right)}_{l}}{{\left(q;q\right)}_{l}})]=(\zeta_{2}r+L)ℵ\leq r.$$

Indeed, $\Theta {ℷ}_{r}\subset {ℷ}_{r}$. Now for $\xi ,\overset{ˆ}{\xi }\in {ℷ}_{r}$ and for any ϑ∈[0,T], we get

$$\left\|\Theta \xi -\Theta \overset{ˆ}{\xi }\right\|\leq \underset{\vartheta \in [0,T]}{\sup}\{\int_{0}^{\vartheta }\frac{{\left(\vartheta -qs\right)}^{\left(\gamma_{1}-1\right)}}{\Gamma_{q}(\gamma_{1})}|\Delta (s,\xi (s))-\Delta (s,\overset{ˆ}{\xi }(s))|\;d_{q}s\;\;+\overset{m}{\sum_{\overset{˙}{ı}=1}}|{ƛ}_{\overset{˙}{ı}}|\int_{0}^{\vartheta }\frac{{\left(\vartheta -qs\right)}^{\left({\gamma_{2}}_{\overset{˙}{ı}}+\gamma_{1}-1\right)}}{\Gamma_{q}({\gamma_{2}}_{\overset{˙}{ı}}+\gamma_{1})}|{\overset{ˆ}{\Delta }}_{\overset{˙}{ı}}(s,\xi (s))-{\overset{ˆ}{\Delta }}_{\overset{˙}{ı}}(s,\overset{ˆ}{\xi }(s))|\;d_{q}s\;\;+\frac{\Gamma_{q}(\gamma_{1}+1)\vartheta }{\left|{\left[\gamma_{1}\right]}_{q}T^{\gamma_{1}-1}-E\right|}(\overset{l}{\sum_{\overset{˙}{ȷ}=1}}|A_{\overset{˙}{ȷ}}|\int_{0}^{{\zeta_{1}}_{\overset{˙}{ȷ}}}\frac{{\left({\zeta_{1}}_{\overset{˙}{ȷ}}-qs\right)}^{\left(2\gamma_{1}-2\right)}}{\Gamma_{q}(2\gamma_{1}-1)}|\Delta (s,\xi (s))-\Delta (s,\overset{ˆ}{\xi }(s))|\;d_{q}s\;\;+\overset{m}{\sum_{\overset{˙}{ı}=1}}\overset{l}{\sum_{\overset{˙}{ȷ}=1}}|A_{\overset{˙}{ȷ}}||{ƛ}_{\overset{˙}{ı}}|\int_{0}^{{\zeta_{1}}_{\overset{˙}{ȷ}}}\frac{{\left({\zeta_{1}}_{\overset{˙}{ȷ}}-qs\right)}^{\left(2\gamma_{1}+{\gamma_{2}}_{\overset{˙}{ı}}-2\right)}}{\Gamma_{q}(2\gamma_{1}+{\gamma_{2}}_{\overset{˙}{ı}}-1)}|{\overset{ˆ}{\Delta }}_{\overset{˙}{ı}}(s,\xi (s))-{\overset{ˆ}{\Delta }}_{\overset{˙}{ı}}(s,\overset{ˆ}{\xi }(s))|\;d_{q}s\;\;+\int_{0}^{T}\frac{{\left(T-qs\right)}^{\left(2\gamma_{1}-3\right)}}{\Gamma_{q}(2\gamma_{1}-2)}|\Delta (s,\xi (s))-\Delta (s,\overset{ˆ}{\xi }(s))|\;d_{q}s\;\;+\overset{m}{\sum_{\overset{˙}{ı}=1}}|{ƛ}_{\overset{˙}{ı}}|\int_{0}^{T}\frac{{\left(T-qs\right)}^{\left(2\gamma_{1}+{\gamma_{2}}_{\overset{˙}{ı}}-3\right)}}{\Gamma_{q}(2\gamma_{1}+{\gamma_{2}}_{\overset{˙}{ı}}-2)}|{\overset{ˆ}{\Delta }}_{\overset{˙}{ı}}(s,\xi (s))-{\overset{ˆ}{\Delta }}_{\overset{˙}{ı}}(s,\overset{ˆ}{\xi }(s))|\;d_{q}s)\}\leq \underset{\vartheta \in [0,T]}{\sup}\{\int_{0}^{\vartheta }\frac{{\left(\vartheta -qs\right)}^{\left(\gamma_{1}-1\right)}}{\Gamma_{q}(\gamma_{1})}\;d_{q}s+\overset{m}{\sum_{\overset{˙}{ı}=1}}|{ƛ}_{\overset{˙}{ı}}|\int_{0}^{\vartheta }\frac{{\left(\vartheta -qs\right)}^{\left({\gamma_{2}}_{\overset{˙}{ı}}+\gamma_{1}-1\right)}}{\Gamma_{q}({\gamma_{2}}_{\overset{˙}{ı}}+\gamma_{1})}\;d_{q}s\;\;+\frac{\Gamma_{q}(\gamma_{1}+1)\vartheta }{\left|{\left[\gamma_{1}\right]}_{q}T^{\gamma_{1}-1}-E\right|}(\overset{l}{\sum_{\overset{˙}{ȷ}=1}}|A_{\overset{˙}{ȷ}}|\int_{0}^{{\zeta_{1}}_{\overset{˙}{ȷ}}}\frac{{\left({\zeta_{1}}_{\overset{˙}{ȷ}}-qs\right)}^{\left(2\gamma_{1}-2\right)}}{\Gamma_{q}(2\gamma_{1}-1)}\;d_{q}s\;\;+\overset{m}{\sum_{\overset{˙}{ı}=1}}\overset{l}{\sum_{\overset{˙}{ȷ}=1}}|A_{\overset{˙}{ȷ}}||{ƛ}_{\overset{˙}{ı}}|\int_{0}^{{\zeta_{1}}_{\overset{˙}{ȷ}}}\frac{{\left({\zeta_{1}}_{\overset{˙}{ȷ}}-qs\right)}^{\left(2\gamma_{1}+{\gamma_{2}}_{\overset{˙}{ı}}-2\right)}}{\Gamma_{q}(2\gamma_{1}+{\gamma_{2}}_{\overset{˙}{ı}}-1)}\;d_{q}s-\int_{0}^{T}\frac{{\left(T-qs\right)}^{\left(2\gamma_{1}-3\right)}}{\Gamma_{q}(2\gamma_{1}-2)}\;d_{q}s\;\;+\overset{m}{\sum_{\overset{˙}{ı}=1}}\frac{\left|{ƛ}_{\overset{˙}{ı}}\right|}{\Gamma_{q}(2\gamma_{1}+{\gamma_{2}}_{\overset{˙}{ı}}-2)}\int_{0}^{T}{\left(T-qs\right)}^{\left(2\gamma_{1}+{\gamma_{2}}_{\overset{˙}{ı}}-3\right)}\;d_{q}s)\}\zeta_{2}\|\xi -\overset{ˆ}{\xi }\|\leq [T^{\gamma_{1}}{\left(1-q\right)}^{\gamma_{1}}\overset{\infty }{\sum_{l=0}}q^{l}\frac{{\left(q^{\gamma_{1}};q\right)}_{l}}{{\left(q;q\right)}_{l}}+\overset{m}{\sum_{\overset{˙}{ı}=1}}|{ƛ}_{\overset{˙}{ı}}|T^{{\gamma_{2}}_{\overset{˙}{ı}}+\gamma_{1}}{\left(1-q\right)}^{{\gamma_{2}}_{\overset{˙}{ı}}+\gamma_{1}}\overset{\infty }{\sum_{l=0}}q^{l}\frac{{\left(q^{{\gamma_{2}}_{\overset{˙}{ı}}+\gamma_{1}};q\right)}_{l}}{{\left(q;q\right)}_{l}}\;\;+\frac{\Gamma_{q}(\gamma_{1}+1)T}{\left|{\left[\gamma_{1}\right]}_{q}T^{\gamma_{1}-1}-E\right|}(\overset{l}{\sum_{\overset{˙}{ȷ}=1}}|A_{\overset{˙}{ȷ}}|{\zeta_{1}}_{\overset{˙}{ȷ}}^{2\gamma_{1}-1}{\left(1-q\right)}^{2\gamma_{1}-1}\overset{\infty }{\sum_{l=0}}q^{l}\frac{{\left(q^{2\gamma_{1}-1};q\right)}_{l}}{{\left(q;q\right)}_{l}}\;\;+\overset{m}{\sum_{\overset{˙}{ı}=1}}\overset{l}{\sum_{\overset{˙}{ȷ}=1}}|A_{\overset{˙}{ȷ}}||{ƛ}_{\overset{˙}{ı}}|{\zeta_{1}}_{\overset{˙}{ȷ}}^{2\gamma_{1}+{\gamma_{2}}_{\overset{˙}{ı}}-1}{\left(1-q\right)}^{2\gamma_{1}+{\gamma_{2}}_{\overset{˙}{ı}}-1}\overset{\infty }{\sum_{l=0}}q^{l}\frac{{\left(q^{2\gamma_{1}+{\gamma_{2}}_{\overset{˙}{ı}}-1};q\right)}_{l}}{{\left(q;q\right)}_{l}}\;\;+T^{2\gamma_{1}-2}{\left(1-q\right)}^{2\gamma_{1}-2}\overset{\infty }{\sum_{l=0}}q^{l}\frac{{\left(q^{2\gamma_{1}-2};q\right)}_{l}}{{\left(q;q\right)}_{l}}\;\;+\overset{m}{\sum_{\overset{˙}{ı}=1}}|{ƛ}_{\overset{˙}{ı}}|T^{2\gamma_{1}+{\gamma_{2}}_{\overset{˙}{ı}}-2}{\left(1-q\right)}^{2\gamma_{1}+{\gamma_{2}}_{\overset{˙}{ı}}-2}\overset{\infty }{\sum_{l=0}}q^{l}\frac{{\left(q^{2\gamma_{1}+{\gamma_{2}}_{\overset{˙}{ı}}-2};q\right)}_{l}}{{\left(q;q\right)}_{l}})]\zeta_{2}\|\xi -\overset{ˆ}{\xi }\|=\zeta_{2}ℵ\|\xi -\overset{ˆ}{\xi }\|,$$

which leads to $\left\|\Theta \xi -\Theta \overset{ˆ}{\xi }\|\leq \zeta_{2}ℵ\|\xi -\overset{ˆ}{\xi }\right\|$. Since $\zeta_{2}ℵ<1$, *Θ* is a contraction mapping. □

Now, in the next theorem, we use Hölder inequality to give another variant of existence and uniqueness result.

Theorem 3.3 *Let* Δ*,* ${\overset{ˆ}{\Delta }}_{\overset{˙}{ı}}\in C([0,T]\times R)$*,* $\overset{˙}{ı}=1,\ldots ,m$ *and assume that:*

(H2) *for each*ϑ∈[0,T]*,*$\left|\Delta (\vartheta ,\xi )-\Delta (\vartheta ,\overset{ˆ}{\xi })|\leq \Psi (\vartheta )|\xi -\overset{ˆ}{\xi }\right|$*and*$\left|{\overset{ˆ}{\Delta }}_{\overset{˙}{ı}}(\vartheta ,\xi )-{\overset{ˆ}{\Delta }}_{\overset{˙}{ı}}(\vartheta ,\overset{ˆ}{\xi })|\leq {Ϝ}_{\overset{˙}{ı}}^{⁎}(\vartheta )(\vartheta )|\xi -\overset{ˆ}{\xi }\right|$*, for*$\xi ,\overset{ˆ}{\xi }\in R$*, where*Ψ(ϑ)*,*℧ı˙∈L1δ([0,T],R+)*,*$\overset{˙}{ı}=1,2,\ldots ,l$*, and*0<δ<1*.*

*If*

(3.6) $$\|\Psi \|{ℵ}_{1}+\overset{m}{\sum_{\overset{˙}{ı}=1}}|{ƛ}_{\overset{˙}{ı}}|\;\|{Ϝ}_{\overset{˙}{ı}}\|\;{ℵ}_{\overset{˙}{ı}+1}<1,$$

*then* (1.1) *has a unique solution, where*

‖θ‖=[∫0T|θ(s¨)|1δdqs¨]δ,θ=Ψ,℧ı˙,ı˙=1,2,…,m,

*and*

(3.7) ℵ1=1Γq(γ1)(∫0T(T−qs)(γ1−1)1−δdqs)1−δ+Γq(γ1+1)T|[γ1]qTγ1−1−E|(∑ȷ˙=1l|Aȷ˙|(∫0ζ1ȷ˙(ζ1ȷ˙−qs)(2γ1−2)1−δΓq(2γ1−1)dqs)1−δ+1Γq(2γ1−2)(∫0T(T−qs)(2γ1−3)1−δdqs)1−δ),

(3.8) ℵı˙+1=1Γq(γ2ı˙+γ1)(∫0T(T−qs)(γ2ı˙+γ1−1)1−δdqs)1−δ+Γq(γ1+1)T|[γ1]qTγ1−1−E|(∑ȷ˙=1l|Aȷ˙|(∫0ζ1ȷ˙(ζ1ȷ˙−qs)(2γ1+γ2ı˙−2)1−δΓq(2γ1+γ2ı˙−1)dqs)1−δ+1Γq(2γ1+γ2ı˙−2)(∫0T(T−qs)(2γ1+γ2ı˙−3)1−δdqs)1−δ),(ı˙=1,2,…,m).

Proof For $\xi ,\overset{ˆ}{\xi }\in F$ and ϑ∈[0,T], by Hölder inequality and using (H2), we have,

‖Θξ−Θξˆ‖≤supϑ∈[0,T]⁡{∫0ϑ(ϑ−qs)(γ1−1)Γq(γ1)Ψ(s)|ξ(s)−ξˆ(s)|dqs+∑ı˙=1m|ƛı˙|∫0ϑ(ϑ−qs)(γ2ı˙+γ1−1)Γq(γ2ı˙+γ1)℧ı˙(s)ξ(s)−ξˆ(s)|dqs+Γq(γ1+1)t|[γ1]qTγ1−1−E|(∑ȷ˙=1k|Aȷ˙|∫0ζ1ȷ˙(ζ1ȷ˙−qs)(2γ1−2)Γq(2γ1−1)Ψ(s)|ξ(s)−ξˆ(s)|dqs+∑ı˙=1m∑ȷ˙=1k|Aȷ˙||ƛı˙|∫0ζ1ȷ˙(ζ1ȷ˙−qs)(2γ1+γ2ı˙−2)Γq(2γ1+γ2ı˙−1)℧ı˙(s)|ξ(s)−ξˆ(s)|dqs+∫0T(T−qs)(2γ1−3)Γq(2γ1−2)Ψ(s)|ξ(s)−ξˆ(s)|dqs+∑ı˙=1m|ƛı˙|∫0T(T−qs)(2γ1+γ2ı˙−3)Γq(2γ1+γ2ı˙−2)℧ı˙(s)|ξ(s)−ξˆ(s)|dqs)}≤supϑ∈[0,T]⁡{1Γq(γ1)(∫0ϑ(ϑ−qs)(γ1−1)1−δdqs)1−δ(∫0ϑΨ(s)1δdqs)δ+1Γq(γ2ı˙+γ1)∑ı˙=1m|ƛı˙|(∫0ϑ(ϑ−qs)(γ2ı˙+γ1−1)1−δdqs)1−δ(∫0ϑ℧ı˙(s)1degdqs)δ+Γq(γ1+1)ϑ|[γ1]qTγ1−1−E|(1Γq(2γ1−1)∑ȷ˙=1k|Aȷ˙|(∫0ζ1ȷ˙(ζ1ȷ˙−qs)(2γ1−2)1−δdqs)1−δ⋅[∫0ζ1ȷ˙u(s)1δdqs]δ+∑ı˙=1m∑ȷ˙=1k|Aȷ˙||ƛı˙|(∫0ζ1ȷ˙(ζ1ȷ˙−qs)(2γ1+γ2ı˙−2)1−δΓq(2γ1+γ2ı˙−1)dqs)1−δ(∫0ζ1ȷ˙℧ı˙(s)1δdqs)δ+1Γq(2γ1−2)(∫0T(T−qs)(2γ1−3)1−δdqs)1−δ(∫0TΨ(s)1δdqs)δ+1Γq(2γ1+γ2ı˙−2)∑ı˙=1m|ƛı˙|(∫0T(T−qs)(2γ1+γ2ı˙−3)1−δdqs)1−δ⋅[∫0T℧ı˙(s)1δdqsdqs]δ)}‖ξ−ξˆ‖=(ℵ1‖℧‖+∑ı˙=1m|ƛı˙|ℵ1+ı˙‖Ϝı˙‖)‖ξ−ξˆ‖.

Therefore, $\left\|\Theta \xi -\Theta \overset{ˆ}{\xi }\|\leq ({ℵ}_{1}\|\Psi \|+\sum_{\overset{˙}{ı}=1}^{m}|{ƛ}_{\overset{˙}{ı}}|{ℵ}_{1+\overset{˙}{ı}}\|{℧}_{\overset{˙}{ı}}\|)\|\xi -\overset{ˆ}{\xi }\right\|$. Thanks to the condition (3.6), *Θ* is a contraction mapping. Hence, by the Banach's FPT *Θ* has a unique FP which is the unique solution of the multi-point BVP (1.1). □

In Theorem 3.4, we employ Leray-Schauder nonlinear alternative [19] to prove the existence of solutions of multi-point BVP (1.1).

Theorem 3.4 *Consider continuous functions* Δ*,* ${\overset{ˆ}{\Delta }}_{\overset{˙}{ı}}:[0,T]\times R\to R$ *and suppose that:*

(H3) *there exist nondecreasing functions*$\Delta ,{\overset{ˆ}{\Delta }}_{\overset{˙}{ı}}:[0,\infty )\to [0,\infty )$*, and*$\overset{ˆ}{b},{\overset{ˆ}{b}}_{\overset{˙}{ı}}\in L^{1}\left([0,T],R^{+}\right)$*s.t.*
  $$\left|\Delta (\vartheta ,\xi )\right|\leq \overset{ˆ}{b}(\vartheta )\Delta (\|\xi \|),\;|{\overset{ˆ}{\Delta }}_{\overset{˙}{ı}}(\vartheta ,\xi )|\leq {\overset{ˆ}{b}}_{\overset{˙}{ı}}(\vartheta )\Delta_{\overset{˙}{ı}}\left(\left\|\xi \right\|\right),$$
  *for each*(ϑ,ξ)∈[0,T]×R*,*$\overset{˙}{ı}=1,\ldots ,m$*;*
(H4) *there exists a constant*N>0*s.t.*
  (3.9) $$N>{\left\|\overset{ˆ}{b}\right\|}_{L^{1}}\Delta \left(N\right)\nabla_{1}+\overset{m}{\sum_{\overset{˙}{ı}=1}}\left|{ƛ}_{\overset{˙}{ı}}\right|{\left\|b_{\overset{˙}{ı}}\right\|}_{L^{1}}\Delta_{\overset{˙}{ı}}\left(N\right)\nabla_{\overset{˙}{ı}+1},$$
  *with*
  (3.10) $$\nabla_{1}=T^{\gamma_{1}}{\left(1-q\right)}^{\gamma_{1}}\overset{\infty }{\sum_{l=0}}q^{l}\frac{{\left(q^{\gamma_{1}};q\right)}_{l}}{{\left(q;q\right)}_{l}}\;\;+\frac{\Gamma_{q}(\gamma_{1}+1)T}{\left|{\left[\gamma_{1}\right]}_{q}T^{\gamma_{1}-1}-E\right|}[\overset{l}{\sum_{\overset{˙}{ȷ}=1}}|A_{\overset{˙}{ȷ}}|{\zeta_{1}}_{\overset{˙}{ȷ}}^{2\gamma_{1}-1}{\left(1-q\right)}^{2\gamma_{1}-1}\overset{\infty }{\sum_{l=0}}q^{l}\frac{{\left(q^{2\gamma_{1}-1};q\right)}_{l}}{{\left(q;q\right)}_{l}}]\;+T^{2\gamma_{1}-2}{\left(1-q\right)}^{2\gamma_{1}-2}\overset{\infty }{\sum_{l=0}}q^{l}\frac{{\left(q^{2\gamma_{1}-2};q\right)}_{l}}{{\left(q;q\right)}_{l}},$$
  (3.11) $$\nabla_{\overset{˙}{ı}+1}=T^{{\gamma_{2}}_{\overset{˙}{ı}}+\gamma_{1}}{\left(1-q\right)}^{{\gamma_{2}}_{\overset{˙}{ı}}+\gamma_{1}}\overset{\infty }{\sum_{l=0}}q^{l}\frac{{\left(q^{{\gamma_{2}}_{\overset{˙}{ı}}+\gamma_{1}};q\right)}_{l}}{{\left(q;q\right)}_{l}}\;\;+\frac{\Gamma_{q}(\gamma_{1}+1)T}{\left|{\left[\gamma_{1}\right]}_{q}T^{\gamma_{1}-1}-E\right|}[\overset{l}{\sum_{\overset{˙}{ȷ}=1}}|A_{\overset{˙}{ȷ}}|{\zeta_{1}}_{\overset{˙}{ȷ}}^{2\gamma_{1}+{\gamma_{2}}_{\overset{˙}{ı}}-1}{\left(1-q\right)}^{2\gamma_{1}+{\gamma_{2}}_{\overset{˙}{ı}}-1}\overset{\infty }{\sum_{l=0}}q^{l}\frac{{\left(q^{2\gamma_{1}+{\gamma_{2}}_{\overset{˙}{ı}}-1};q\right)}_{l}}{{\left(q;q\right)}_{l}}]\;+T^{2\gamma_{1}+{\gamma_{2}}_{\overset{˙}{ı}}-2}{\left(1-q\right)}^{2\gamma_{1}+{\gamma_{2}}_{\overset{˙}{ı}}-2}\overset{\infty }{\sum_{l=0}}q^{l}\frac{{\left(q^{2\gamma_{1}+{\gamma_{2}}_{\overset{˙}{ı}}-2};q\right)}_{l}}{{\left(q;q\right)}_{l}}.$$

*Then the multi-point* BVP (1.1) *has at least one solution on* [0,T]*.*

Proof Consider Θ:F→F is expressed by (3.4). Take bounded set ${ℷ}_{r}=\{\xi \in F\;:\;\left\|\xi \right\|\leq r\}$ in F for r>0. Then, for ϑ∈[0,T] and (H3), we have

$$|\Theta \xi \left(\vartheta \right)|\leq \int_{0}^{\vartheta }\frac{{\left(\vartheta -qs\right)}^{\left(\gamma_{1}-1\right)}}{\Gamma_{q}(\gamma_{1})}\overset{ˆ}{b}\left(s\right)\Delta \left(\left\|\xi \right\|\right)\;d_{q}s\;\;+\overset{m}{\sum_{\overset{˙}{ı}=1}}|{ƛ}_{\overset{˙}{ı}}|\int_{0}^{\vartheta }\frac{{\left(\vartheta -qs\right)}^{\left({\gamma_{2}}_{\overset{˙}{ı}}+\gamma_{1}-1\right)}}{\Gamma_{q}({\gamma_{2}}_{\overset{˙}{ı}}+\gamma_{1})}{\overset{ˆ}{b}}_{\overset{˙}{ı}}\left(s\right)\Delta_{\overset{˙}{ı}}\left(\left\|\xi \right\|\right)\;d_{q}s\;\;+\frac{\Gamma_{q}(\gamma_{1}+1)\vartheta }{\left|{\left[\gamma_{1}\right]}_{q}T^{\gamma_{1}-1}-E\right|}(\overset{l}{\sum_{\overset{˙}{ȷ}=1}}|A_{\overset{˙}{ȷ}}|\int_{0}^{{\zeta_{1}}_{\overset{˙}{ȷ}}}\frac{{\left({\zeta_{1}}_{\overset{˙}{ȷ}}-qs\right)}^{\left(2\gamma_{1}-2\right)}}{\Gamma_{q}(2\gamma_{1}-1)}\overset{ˆ}{b}\left(s\right)\Delta \left(\left\|\xi \right\|\right)\;d_{q}s\;\;+\overset{m}{\sum_{\overset{˙}{ı}=1}}\overset{l}{\sum_{\overset{˙}{ȷ}=1}}|A_{\overset{˙}{ȷ}}||{ƛ}_{\overset{˙}{ı}}|\int_{0}^{{\zeta_{1}}_{\overset{˙}{ȷ}}}\frac{{\left({\zeta_{1}}_{\overset{˙}{ȷ}}-qs\right)}^{\left(2\gamma_{1}+{\gamma_{2}}_{\overset{˙}{ı}}-2\right)}}{\Gamma_{q}(2\gamma_{1}+{\gamma_{2}}_{\overset{˙}{ı}}-1)}{\overset{ˆ}{b}}_{\overset{˙}{ı}}\left(s\right)\Delta_{\overset{˙}{ı}}\left(\left\|\xi \right\|\right)\;d_{q}s\;\;+\int_{0}^{T}\frac{{\left(T-qs\right)}^{\left(2\gamma_{1}-3\right)}}{\Gamma_{q}(2\gamma_{1}-2)}\overset{ˆ}{b}\left(s\right)\Delta \left(\left\|\xi \right\|\right)\;d_{q}s\;\;+\overset{m}{\sum_{\overset{˙}{ı}=1}}|{ƛ}_{\overset{˙}{ı}}|\int_{0}^{T}\frac{{\left(T-qs\right)}^{\left(2\gamma_{1}+{\gamma_{2}}_{\overset{˙}{ı}}-3\right)}}{\Gamma_{q}(2\gamma_{1}+{\gamma_{2}}_{\overset{˙}{ı}}-2)}{\overset{ˆ}{b}}_{\overset{˙}{ı}}\left(s\right)\Delta_{\overset{˙}{ı}}\left(\left\|\xi \right\|\right)\;d_{q}s).$$

Consequently,

$$\|\Theta \xi \|\leq {\left\|\overset{ˆ}{b}\right\|}_{L^{1}}\Delta \left(r\right)(T^{\gamma_{1}}{\left(1-q\right)}^{\gamma_{1}}\overset{\infty }{\sum_{l=0}}q^{l}\frac{{\left(q^{\gamma_{1}};q\right)}_{l}}{{\left(q;q\right)}_{l}}\;\;+\frac{\Gamma_{q}(\gamma_{1}+1)T}{\left|{\left[\gamma_{1}\right]}_{q}T^{\gamma_{1}-1}-E\right|}(\overset{l}{\sum_{\overset{˙}{ȷ}=1}}|A_{\overset{˙}{ȷ}}|{\zeta_{1}}_{\overset{˙}{ȷ}}^{2\gamma_{1}-1}{\left(1-q\right)}^{2\gamma_{1}-1}\overset{\infty }{\sum_{l=0}}q^{l}\frac{{\left(q^{2\gamma_{1}-1};q\right)}_{l}}{{\left(q;q\right)}_{l}})\;+T^{2\gamma_{1}-1}{\left(1-q\right)}^{2\gamma_{1}-1}\overset{\infty }{\sum_{l=0}}q^{l}\frac{{\left(q^{2\gamma_{1}-1};q\right)}_{l}}{{\left(q;q\right)}_{l}})\;+\overset{m}{\sum_{\overset{˙}{ı}=1}}\left|{ƛ}_{\overset{˙}{ı}}\right|{\left\|{\overset{ˆ}{b}}_{\overset{˙}{ı}}\right\|}_{L^{1}}\Delta_{\overset{˙}{ı}}\left(r\right)(T^{{\gamma_{2}}_{\overset{˙}{ı}}+\gamma_{1}}{\left(1-q\right)}^{{\gamma_{2}}_{\overset{˙}{ı}}+\gamma_{1}}\overset{\infty }{\sum_{l=0}}q^{l}\frac{{\left(q^{{\gamma_{2}}_{\overset{˙}{ı}}+\gamma_{1}};q\right)}_{l}}{{\left(q;q\right)}_{l}}\;\;+\frac{\Gamma_{q}(\gamma_{1}+1)T}{\left|{\left[\gamma_{1}\right]}_{q}T^{\gamma_{1}-1}-E\right|}(\overset{l}{\sum_{\overset{˙}{ȷ}=1}}|A_{\overset{˙}{ȷ}}|{\zeta_{1}}_{\overset{˙}{ȷ}}^{2\gamma_{1}+{\gamma_{2}}_{\overset{˙}{ı}}-1}{\left(1-q\right)}^{2\gamma_{1}+{\gamma_{2}}_{\overset{˙}{ı}}-1}\overset{\infty }{\sum_{l=0}}q^{l}\frac{{\left(q^{2\gamma_{1}+{\gamma_{2}}_{\overset{˙}{ı}}-1};q\right)}_{l}}{{\left(q;q\right)}_{l}})\;+T^{2\gamma_{1}+{\gamma_{2}}_{\overset{˙}{ı}}-2}{\left(1-q\right)}^{2\gamma_{1}+{\gamma_{2}}_{\overset{˙}{ı}}-2}\overset{\infty }{\sum_{l=0}}q^{l}\frac{{\left(q^{2\gamma_{1}+{\gamma_{2}}_{\overset{˙}{ı}}-2};q\right)}_{l}}{{\left(q;q\right)}_{l}})={\left\|\overset{ˆ}{b}\right\|}_{L^{1}}\Delta (r)\nabla_{1}+\overset{m}{\sum_{\overset{˙}{ı}=1}}|{ƛ}_{\overset{˙}{ı}}|{\left\|{\overset{ˆ}{b}}_{\overset{˙}{ı}}\right\|}_{L^{1}}\Delta_{\overset{˙}{ı}}(r)\nabla_{\overset{˙}{ı}+1}=K.$$

Therefore ‖Θξ‖≤K. Hence, *Θ* maps bounded sets into bounded sets in F. Next, we show that *Θ* maps bounded sets into equicontinuous sets of F. Let $\vartheta_{1},\vartheta_{2}\in [0,T]$, $\vartheta_{1}<\vartheta_{2}$ and $\xi \in {ℷ}_{r}$. Then, we obtain

$$\left|\Theta \xi (\vartheta_{2})-\Theta \xi (\vartheta_{1})\right|\leq \int_{0}^{\vartheta_{1}}\frac{{\left(\vartheta_{2}-qs\right)}^{\gamma_{1}-1}-{\left(\vartheta_{1}-qs\right)}^{\gamma_{1}-1}}{\Gamma_{q}(\gamma_{1})}|\Delta (s,\xi (s))|\;d_{q}s\;\;+\int_{\vartheta_{1}}^{\vartheta_{2}}\frac{{\left(\vartheta_{2}-qs\right)}^{\gamma_{1}-1}}{\Gamma_{q}(\gamma_{1})}|\Delta (s,\xi (s))|\;d_{q}s\;\;+\overset{m}{\sum_{\overset{˙}{ı}=1}}\left|{ƛ}_{\overset{˙}{ı}}\right|\int_{0}^{\vartheta_{1}}\frac{{\left(\vartheta_{2}-qs\right)}^{{\gamma_{2}}_{\overset{˙}{ı}}+\gamma_{1}-1}-{\left(\vartheta_{1}-qs\right)}^{{\gamma_{2}}_{\overset{˙}{ı}}+\gamma_{1}-1}}{\Gamma_{q}({\gamma_{2}}_{\overset{˙}{ı}}+\gamma_{1})}|{\overset{ˆ}{\Delta }}_{\overset{˙}{ı}}(s,\xi (s))|\;d_{q}s\;\;+\overset{m}{\sum_{\overset{˙}{ı}=1}}\left|{ƛ}_{\overset{˙}{ı}}\right|\int_{\vartheta_{1}}^{\vartheta_{2}}\frac{{\left(\vartheta_{2}-qs\right)}^{{\gamma_{2}}_{\overset{˙}{ı}}+\gamma_{1}-1}}{\Gamma_{q}({\gamma_{2}}_{\overset{˙}{ı}}+\gamma_{1})}|{\overset{ˆ}{\Delta }}_{\overset{˙}{ı}}(s,\xi (s))|\;d_{q}s\;\;+\frac{\Gamma_{q}(\gamma_{1}+1)(\vartheta_{2}-\vartheta_{1})}{\left|{\left[\gamma_{1}\right]}_{q}T^{\gamma_{1}-1}-E\right|}[\overset{l}{\sum_{\overset{˙}{ȷ}=1}}|A_{\overset{˙}{ȷ}}|\int_{0}^{{\zeta_{1}}_{\overset{˙}{ȷ}}}\frac{{\left({\zeta_{1}}_{\overset{˙}{ȷ}}-qs\right)}^{\left(2\gamma_{1}-2\right)}}{\Gamma_{q}(2\gamma_{1}-1)}|\Delta (s,\xi (s))|\;d_{q}s\;\;+\overset{m}{\sum_{\overset{˙}{ı}=1}}\overset{l}{\sum_{\overset{˙}{ȷ}=1}}|A_{\overset{˙}{ȷ}}||{ƛ}_{\overset{˙}{ı}}|\int_{0}^{{\zeta_{1}}_{\overset{˙}{ȷ}}}\frac{{\left({\zeta_{1}}_{\overset{˙}{ȷ}}-qs\right)}^{\left(2\gamma_{1}+{\gamma_{2}}_{\overset{˙}{ı}}-2\right)}}{\Gamma_{q}(2\gamma_{1}+{\gamma_{2}}_{\overset{˙}{ı}}-1)}|{\overset{ˆ}{\Delta }}_{\overset{˙}{ı}}(s,\xi (s))|\;d_{q}s\;\;+\int_{0}^{T}\frac{{\left(T-qs\right)}^{\left(2\gamma_{1}-3\right)}}{\Gamma_{q}(2\gamma_{1}-2)}|\Delta (s,\xi (s))|\;d_{q}s\;\;+\overset{m}{\sum_{\overset{˙}{ı}=1}}|{ƛ}_{\overset{˙}{ı}}|\int_{0}^{T}\frac{{\left(T-qs\right)}^{\left(2\gamma_{1}+{\gamma_{2}}_{\overset{˙}{ı}}-3\right)}}{\Gamma_{q}(2\gamma_{1}+{\gamma_{2}}_{\overset{˙}{ı}}-2)}|{\overset{ˆ}{\Delta }}_{\overset{˙}{ı}}(s,\xi (s))|\;d_{q}s]\leq \int_{0}^{\vartheta_{1}}\frac{{\left(\vartheta_{2}-qs\right)}^{\gamma_{1}-1}-{\left(\vartheta_{1}-qs\right)}^{\gamma_{1}-1}}{\Gamma_{q}(\gamma_{1})}\overset{ˆ}{b}\left(s\right)\Delta \left(r\right)\;d_{q}s\;\;+\int_{\vartheta_{1}}^{\vartheta_{2}}\frac{{\left(\vartheta_{2}-qs\right)}^{\gamma_{1}-1}}{\Gamma_{q}(\gamma_{1})}\overset{ˆ}{b}\left(s\right)\Delta \left(r\right)\;d_{q}s\;\;+\overset{m}{\sum_{\overset{˙}{ı}=1}}\left|{ƛ}_{\overset{˙}{ı}}\right|\int_{0}^{\vartheta_{1}}\frac{{\left(\vartheta_{2}-qs\right)}^{{\gamma_{2}}_{\overset{˙}{ı}}+\gamma_{1}-1}-{\left(\vartheta_{1}-qs\right)}^{{\gamma_{2}}_{\overset{˙}{ı}}+\gamma_{1}-1}}{\Gamma_{q}({\gamma_{2}}_{\overset{˙}{ı}}+\gamma_{1})}{\overset{ˆ}{b}}_{\overset{˙}{ı}}(s)\Delta_{\overset{˙}{ı}}\left(r\right)\;d_{q}s\;\;+\overset{m}{\sum_{\overset{˙}{ı}=1}}\left|{ƛ}_{\overset{˙}{ı}}\right|\int_{\vartheta_{1}}^{\vartheta_{2}}\frac{{\left(\vartheta_{2}-qs\right)}^{{\gamma_{2}}_{\overset{˙}{ı}}+\gamma_{1}-1}}{\Gamma_{q}({\gamma_{2}}_{\overset{˙}{ı}}+\gamma_{1})}{\overset{ˆ}{b}}_{\overset{˙}{ı}}(s)\Delta_{\overset{˙}{ı}}(r)\;d_{q}s\;\;+\frac{\Gamma_{q}(\gamma_{1}+1)(\vartheta_{2}-\vartheta_{1})}{\left|{\left[\gamma_{1}\right]}_{q}T^{\gamma_{1}-1}-E\right|}[\overset{l}{\sum_{\overset{˙}{ȷ}=1}}|A_{\overset{˙}{ȷ}}|\int_{0}^{{\zeta_{1}}_{\overset{˙}{ȷ}}}\frac{{\left({\zeta_{1}}_{\overset{˙}{ȷ}}-qs\right)}^{\left(2\gamma_{1}-2\right)}}{\Gamma_{q}(2\gamma_{1}-1)}\overset{ˆ}{b}(s)\Delta (r)\;d_{q}s\;\;+\overset{m}{\sum_{\overset{˙}{ı}=1}}\overset{l}{\sum_{\overset{˙}{ȷ}=1}}|A_{\overset{˙}{ȷ}}||{ƛ}_{\overset{˙}{ı}}|\int_{0}^{{\zeta_{1}}_{\overset{˙}{ȷ}}}\frac{{\left({\zeta_{1}}_{\overset{˙}{ȷ}}-qs\right)}^{\left(2\gamma_{1}+{\gamma_{2}}_{\overset{˙}{ı}}-2\right)}}{\Gamma_{q}(2\gamma_{1}+{\gamma_{2}}_{\overset{˙}{ı}}-1)}{\overset{ˆ}{b}}_{\overset{˙}{ı}}(s)\Delta_{\overset{˙}{ı}}(r)\;d_{q}s\;\;+\int_{0}^{T}\frac{{\left(T-qs\right)}^{\left(2\gamma_{1}-3\right)}}{\Gamma_{q}(2\gamma_{1}-2)}\overset{ˆ}{b}\left(s\right)\Delta \left(r\right)\;d_{q}\lambda \;\;+\overset{m}{\sum_{\overset{˙}{ı}=1}}|{ƛ}_{\overset{˙}{ı}}|\int_{0}^{T}\frac{{\left(T-q\lambda \right)}^{\left(2\gamma_{1}+{\gamma_{2}}_{\overset{˙}{ı}}-3\right)}}{\Gamma_{q}(2\gamma_{1}+{\gamma_{2}}_{\overset{˙}{ı}}-2)}{\overset{ˆ}{b}}_{\overset{˙}{ı}}(\lambda )\Delta_{\overset{˙}{ı}}\left(r\right)\;d_{q}\lambda ]\leq {\left\|\overset{ˆ}{b}\right\|}_{L^{1}}\Delta (r)(\vartheta_{2}^{\gamma_{1}}-\vartheta_{1}^{\gamma_{1}}){\left(1-q\right)}^{\gamma_{1}}\overset{\infty }{\sum_{l=0}}q^{l}\frac{{\left(q^{\gamma_{1}};q\right)}_{l}}{{\left(q;q\right)}_{l}}\;\;+\overset{m}{\sum_{\overset{˙}{ı}=1}}\left|{ƛ}_{\overset{˙}{ı}}\right|{\left\|{\overset{ˆ}{b}}_{\overset{˙}{ı}}\right\|}_{L^{1}}\Delta_{\overset{˙}{ı}}\left(r\right)(\vartheta_{2}^{{\gamma_{2}}_{\overset{˙}{ı}}+\gamma_{1}}-\vartheta_{1}^{{\gamma_{2}}_{\overset{˙}{ı}}+\gamma_{1}}){\left(1-q\right)}^{\gamma_{1}}\overset{\infty }{\sum_{l=0}}q^{l}\frac{{\left(q^{{\gamma_{2}}_{\overset{˙}{ı}}+\gamma_{1}};q\right)}_{l}}{{\left(q;q\right)}_{l}}\;\;+\frac{\Gamma_{q}(\gamma_{1}+1)(\vartheta_{2}-\vartheta_{1})}{\left|{\left[\gamma_{1}\right]}_{q}T^{\gamma_{1}-1}-E\right|}[\overset{l}{\sum_{\overset{˙}{ȷ}=1}}{\left\|\overset{ˆ}{b}\right\|}_{L^{1}}\Delta (r)|A_{\overset{˙}{ȷ}}|{\zeta_{1}}_{\overset{˙}{ȷ}}^{2\gamma_{1}-1}{\left(1-q\right)}^{2\gamma_{1}-1}\overset{\infty }{\sum_{l=0}}q^{l}\frac{{\left(q^{2\gamma_{1}-1};q\right)}_{l}}{{\left(q;q\right)}_{l}}\;\;+\overset{m}{\sum_{\overset{˙}{ı}=1}}\overset{l}{\sum_{\overset{˙}{ȷ}=1}}{\left\|{\overset{ˆ}{b}}_{\overset{˙}{ı}}\right\|}_{L^{1}}\Delta_{i}\left(r\right)|A_{\overset{˙}{ȷ}}||{ƛ}_{\overset{˙}{ı}}|{\zeta_{1}}_{\overset{˙}{ȷ}}^{2\gamma_{1}+{\gamma_{2}}_{\overset{˙}{ı}}-1}{\left(1-q\right)}^{2\gamma_{1}+{\gamma_{2}}_{\overset{˙}{ı}}-1}\overset{\infty }{\sum_{l=0}}q^{l}\frac{{\left(q^{2\gamma_{1}+{\gamma_{2}}_{\overset{˙}{ı}}-1};q\right)}_{l}}{{\left(q;q\right)}_{l}}\;\;+T^{2\gamma_{1}-2}{\left\|\overset{ˆ}{b}\right\|}_{L^{1}}\Delta (r){\left(1-q\right)}^{2\gamma_{1}-2}\overset{\infty }{\sum_{l=0}}q^{l}\frac{{\left(q^{2\gamma_{1}-2};q\right)}_{l}}{{\left(q;q\right)}_{l}}\;\;+\overset{m}{\sum_{\overset{˙}{ı}=1}}|{ƛ}_{\overset{˙}{ı}}|T^{2\gamma_{1}+{\gamma_{2}}_{\overset{˙}{ı}}-2}{\left\|{\overset{ˆ}{b}}_{\overset{˙}{ı}}\right\|}_{L^{1}}\Delta_{\overset{˙}{ı}}\left(r\right){\left(1-q\right)}^{2\gamma_{1}+{\gamma_{2}}_{\overset{˙}{ı}}-2}\overset{\infty }{\sum_{l=0}}q^{l}\frac{{\left(q^{2\gamma_{1}+{\gamma_{2}}_{\overset{˙}{ı}}-2};q\right)}_{l}}{{\left(q;q\right)}_{l}}].$$

Clearly, this inequality tends to zero independently of $\xi \in {ℷ}_{r}$ as $\vartheta_{2}-\vartheta_{1}\to 0$ and so, Arzelà-Ascoli theorem implies that Θ:F→F is completely continuous. Now, we can conclude the result by employing the Leray-Schauder nonlinear alternative for single valued maps. Consider the equation ξ=χΘξ for 0<χ<1 and assume that *ξ* be a solution. Taking the computations in proving that *Θ* is bounded, we obtain,

$$\left\|\xi \right\|=\left\|\chi \Theta \xi \right\|\leq {\left\|\overset{ˆ}{b}\right\|}_{L^{1}}\Delta \left(r\right)[T^{\gamma_{1}}{\left(1-q\right)}^{\gamma_{1}}\overset{\infty }{\sum_{l=0}}q^{l}\frac{{\left(q^{\gamma_{1}};q\right)}_{l}}{{\left(q;q\right)}_{l}}\;\;+\frac{\Gamma_{q}(\gamma_{1}+1)T}{\left|{\left[\gamma_{1}\right]}_{q}T^{\gamma_{1}-1}-E\right|}[\overset{l}{\sum_{\overset{˙}{ȷ}=1}}|A_{\overset{˙}{ȷ}}|{\zeta_{1}}_{\overset{˙}{ȷ}}^{2\gamma_{1}-1}{\left(1-q\right)}^{2\gamma_{1}-1}\overset{\infty }{\sum_{l=0}}q^{l}\frac{{\left(q^{2\gamma_{1}-1};q\right)}_{l}}{{\left(q;q\right)}_{l}}]\;+T^{2\gamma_{1}-1}{\left(1-q\right)}^{2\gamma_{1}-1}\overset{\infty }{\sum_{l=0}}q^{l}\frac{{\left(q^{2\gamma_{1}-1};q\right)}_{l}}{{\left(q;q\right)}_{l}}]\;+\overset{m}{\sum_{\overset{˙}{ı}=1}}\left|{ƛ}_{\overset{˙}{ı}}\right|{\left\|{\overset{ˆ}{b}}_{\overset{˙}{ı}}\right\|}_{L^{1}}\Delta_{\overset{˙}{ı}}(r)[T^{{\gamma_{2}}_{\overset{˙}{ı}}+\gamma_{1}}{\left(1-q\right)}^{{\gamma_{2}}_{\overset{˙}{ı}}+\gamma_{1}}\overset{\infty }{\sum_{l=0}}q^{l}\frac{{\left(q^{{\gamma_{2}}_{\overset{˙}{ı}}+\gamma_{1}};q\right)}_{l}}{{\left(q;q\right)}_{l}}\;\;+\frac{\Gamma_{q}(\gamma_{1}+1)T}{\left|{\left[\gamma_{1}\right]}_{q}T^{\gamma_{1}-1}-E\right|}[\overset{l}{\sum_{\overset{˙}{ȷ}=1}}|A_{\overset{˙}{ȷ}}|{\zeta_{1}}_{\overset{˙}{ȷ}}^{2\gamma_{1}+{\gamma_{2}}_{\overset{˙}{ı}}-1}{\left(1-q\right)}^{2\gamma_{1}+{\gamma_{2}}_{\overset{˙}{ı}}-1}\overset{\infty }{\sum_{l=0}}q^{l}\frac{{\left(q^{2\gamma_{1}+{\gamma_{2}}_{\overset{˙}{ı}}-1};q\right)}_{l}}{{\left(q;q\right)}_{l}}]\;+T^{2\gamma_{1}+{\gamma_{2}}_{\overset{˙}{ı}}-2}{\left(1-q\right)}^{2\gamma_{1}+{\gamma_{2}}_{\overset{˙}{ı}}-2}\overset{\infty }{\sum_{l=0}}q^{l}\frac{{\left(q^{2\gamma_{1}+{\gamma_{2}}_{\overset{˙}{ı}}-2};q\right)}_{l}}{{\left(q;q\right)}_{l}}].$$

Therefore,

$$\left\|\xi \right\|\leq {\left\|\overset{ˆ}{b}\right\|}_{L^{1}}\Delta (r)\nabla_{1}+\overset{m}{\sum_{\overset{˙}{ı}=1}}\left|{ƛ}_{\overset{˙}{ı}}\right|\;{\left\|{\overset{ˆ}{b}}_{\overset{˙}{ı}}\right\|}_{L^{1}}\Delta_{\overset{˙}{ı}}(r)\nabla_{\overset{˙}{ı}+1}.$$

By (H4), there exists *N* s.t. N≠‖ξ‖. Let us set Ω={ξ∈F:‖ξ‖<N}. This implies that $\Theta :\bar{\Omega }\to F$ is continuous and completely continuous. From the choice of *χ*, there is no ξ∈∂Ω s.t. ξ=χΘξ for some 0<χ<1. Consequently, by the nonlinear alternative of Leray-Schauder's type, we deduce that *Θ* has a FP $\xi \in \bar{\Omega }$ which is a solution of the multi-point BVP (1.1). □

We also prove the existence of solutions of multi-point BVP (1.1) by employing Leray-Schauder degree.

Theorem 3.5 *For* Δ*,* ${\overset{ˆ}{\Delta }}_{\overset{˙}{ı}}\in C([0,T]\times R)$*,* $\overset{˙}{ı}=1,\ldots ,m$*, suppose that*

(H5) *there exist constants*
  $$0\leq \overset{˜}{a}<{\left[\nabla_{1}+\overset{m}{\sum_{\overset{˙}{ı}=1}}\left|{ƛ}_{\overset{˙}{ı}}\right|\nabla_{\overset{˙}{ı}+1}\right]}^{-1},\;{\overset{˜}{M}}_{\overset{˙}{ı}}>0,\;(\overset{˙}{ı}=1,\ldots ,m+1),$$
  *with*
  $$|\Delta \left(\vartheta ,\xi \right)|\leq {\overset{˜}{a}}_{1}(|\xi |)+{\overset{˜}{M}}_{1},\;|{\overset{ˆ}{\Delta }}_{\overset{˙}{ı}}\left(\vartheta ,\xi \right)|\leq {\overset{˜}{a}}_{\overset{˙}{ı}+1}\left(\left|\xi \right|\right)+{\overset{˜}{M}}_{\overset{˙}{ı}+1},$$
  *here*$\overset{˙}{ı}=1,\ldots ,m$*,*(ϑ,ξ)∈[0,T]×R*, and*$\overset{˜}{a}=\max\{{\overset{˜}{a}}_{\overset{˙}{ı}}\;:\;\overset{˙}{ı}=1,\ldots ,m+1\}$*,*$\overset{˜}{M}=\max\{{\overset{˜}{M}}_{\overset{˙}{ı}}\;:\;\overset{˙}{ı}=1,\ldots ,m+1\}$*.*

*Then the multi-point* BVP (1.1) *has at least one solution on* [0,T]*.*

Proof We define Θ:F→F as in (3.4) and consider the FP equation ξ=Θξ. We shall prove that there exists a FP ξ∈F satisfying (1.1). In this case, show that $\Theta :{\bar{B}}_{r}\to F$ satisfies

(3.12) $$\xi \neq \delta \Theta \xi ,\;\forall \;\left(\xi ,\delta \right)\in \partial {ℷ}_{r}\times \left[0,1\right],$$

where

$${ℷ}_{r}:=\{\xi \in F\;:\;\max_{\vartheta \in [0,T]}\left|\xi \left(\vartheta \right)\right|<r,\;r>0\}.$$

We define $S\left(\zeta_{2},\xi \right)=\delta \Theta \xi$ for (ξ,δ)∈F×[0,1]. As shown in Theorem 3.4, the operator *Θ* is continuous, uniformly bounded, and equicontinuous. The Arzelà-Ascoli theorem implies that a continuous map $s_{\delta }$ is expressed by $s_{\delta }=\xi -S\left(\zeta_{2},\xi \right)=\xi -\delta \Theta \xi$, is completely continuous. If (3.12) holds, then the following Leray-Schauder degrees are well defined and by the homotopy invariance of topological degree, it follows that

$$\deg\left(s_{\delta },{ℷ}_{r},0\right)=\deg\left(I-\delta \Theta ,{ℷ}_{r},0\right)=\deg\left(s_{1},{ℷ}_{r},0\right)=\deg\left(s_{0},{ℷ}_{r},0\right)=\deg\left(I,{ℷ}_{r},0\right)=1\neq 0,\;0\in {ℷ}_{r},$$

with the identity operator *I*. By the nonzero property of Leray-Schauder's degree, $s_{1}(\xi )=\xi -\Theta \xi =0$ for at least one $\xi \in {ℷ}_{r}$. In order to prove (3.12), we assume that ξ=δΘξ for some δ∈[0,1] and ϑ∈[0,T]. Then

$$\left|\xi \left(\vartheta \right)\right|=\left|\delta \Theta \xi \left(\vartheta \right)\right|\leq \left(\overset{˜}{a}\left|\xi \left(\vartheta \right)\right|+\overset{˜}{M}\right)[T^{\gamma_{1}}{\left(1-q\right)}^{\gamma_{1}}\overset{\infty }{\sum_{l=0}}q^{l}\frac{{\left(q^{\gamma_{1}};q\right)}_{l}}{{\left(q;q\right)}_{l}}\;\;+\frac{\Gamma_{q}(\gamma_{1}+1)T}{\left|{\left[\gamma_{1}\right]}_{q}T^{\gamma_{1}-1}-E\right|}(\overset{l}{\sum_{\overset{˙}{ȷ}=1}}|A_{\overset{˙}{ȷ}}|{\zeta_{1}}_{\overset{˙}{ȷ}}^{2\gamma_{1}-1}{\left(1-q\right)}^{2\gamma_{1}-1}\overset{\infty }{\sum_{l=0}}q^{l}\frac{{\left(q^{2\gamma_{1}-1};q\right)}_{l}}{{\left(q;q\right)}_{l}})\;+T^{2\gamma_{1}-1}{\left(1-q\right)}^{2\gamma_{1}-1}\overset{\infty }{\sum_{l=0}}q^{l}\frac{{\left(q^{2\gamma_{1}-1};q\right)}_{l}}{{\left(q;q\right)}_{l}}\;\;+\overset{m}{\sum_{\overset{˙}{ı}=1}}|{ƛ}_{\overset{˙}{ı}}|(T^{{\gamma_{2}}_{\overset{˙}{ı}}+\gamma_{1}}{\left(1-q\right)}^{{\gamma_{2}}_{\overset{˙}{ı}}+\gamma_{1}}\overset{\infty }{\sum_{l=0}}q^{l}\frac{{\left(q^{{\gamma_{2}}_{\overset{˙}{ı}}+\gamma_{1}};q\right)}_{l}}{{\left(q;q\right)}_{l}}\;\;+\frac{\Gamma_{q}(\gamma_{1}+1)T}{\left|{\left[\gamma_{1}\right]}_{q}T^{\gamma_{1}-1}-E\right|}(\overset{l}{\sum_{\overset{˙}{ȷ}=1}}|A_{\overset{˙}{ȷ}}|{\zeta_{1}}_{\overset{˙}{ȷ}}^{2\gamma_{1}+{\gamma_{2}}_{\overset{˙}{ı}}-1}{\left(1-q\right)}^{2\gamma_{1}+{\gamma_{2}}_{\overset{˙}{ı}}-1}\overset{\infty }{\sum_{l=0}}q^{l}\frac{{\left(q^{2\gamma_{1}+{\gamma_{2}}_{\overset{˙}{ı}}-1};q\right)}_{l}}{{\left(q;q\right)}_{l}})\;+T^{2\gamma_{1}+{\gamma_{2}}_{\overset{˙}{ı}}-2}{\left(1-q\right)}^{2\gamma_{1}+{\gamma_{2}}_{\overset{˙}{ı}}-2}\overset{\infty }{\sum_{l=0}}q^{l}\frac{{\left(q^{2\gamma_{1}+{\gamma_{2}}_{\overset{˙}{ı}}-2};q\right)}_{l}}{{\left(q;q\right)}_{l}})]=\left(\overset{˜}{a}\left|\xi \left(\vartheta \right)\right|+\overset{˜}{M}\right)[\nabla_{1}+\overset{m}{\sum_{\overset{˙}{ı}=1}}\left|{ƛ}_{\overset{˙}{ı}}\right|\nabla_{\overset{˙}{ı}+1}].$$

Taking norm $\sup_{\vartheta \in [0,T]}\left|\xi \left(\vartheta \right)\right|=\left\|\xi \right\|$, we get

$$\left\|\xi \right\|\leq \left(\overset{˜}{a}\left\|\xi \right\|+\overset{˜}{M}\right)[\nabla_{1}+\sum_{\overset{˙}{ı}=1}^{l}\left|{ƛ}_{\overset{˙}{ı}}\right|\nabla_{\overset{˙}{ı}+1}],$$

which implies that

$$\left\|\xi \right\|\leq \frac{\overset{˜}{M}}{1-\overset{˜}{a}\left(\nabla_{1}+\sum_{\overset{˙}{ı}=1}^{l}\left|{ƛ}_{\overset{˙}{ı}}\right|\nabla_{\overset{˙}{ı}+1}\right)}[(\nabla_{1}+\sum_{\overset{˙}{ı}=1}^{l}\left|{ƛ}_{\overset{˙}{ı}}\right|\nabla_{\overset{˙}{ı}+1})].$$

If

$$r=\overset{˜}{M}(\nabla_{1}+\overset{m}{\sum_{\overset{˙}{ı}=1}}\left|{ƛ}_{\overset{˙}{ı}}\right|\nabla_{\overset{˙}{ı}+1}){\left[1-\overset{˜}{a}(\nabla_{1}+\overset{m}{\sum_{\overset{˙}{ı}=1}}\left|{ƛ}_{\overset{˙}{ı}}\right|\nabla_{\overset{˙}{ı}+1})\right]}^{-1}+1,$$

then inequality (3.12) holds. □

## 4 Illustrative applications

To explain our main findings, we use the following examples. In the first example, we examine the results of changes in the order of the derivative $\gamma_{1}$. To perform calculations, successful algorithms are used in [10].

All the experiments are carried out in MATLAB Ver. 8.5.0.197613 (R2015a) on a computer equipped with a CPU AMD Athlon(tm) II X2245 at 2.90 GHz running under the operating system Windows 7.

Example 4.1 Let us consider the following multi-point BVP,

(4.1) {D0.5γ1Cξ(ϑ)=Δ(ϑ,ξ(ϑ))+∑ı˙=1mƛı˙I0.5γ2ı˙Δˆı˙(ϑ,ξ(ϑ)),m=3,I0.52−γ1ξ(0)=0,D0.5γ1ξ(1)=∑ȷ˙=1lAȷ˙I0.5γ1−1ξ(ζ1ȷ˙),l=2,

for ϑ∈[0,T]=[0,1], with T=1, $q=\frac{1}{2}$ and three values of

$$\gamma_{1}\in \left\{\frac{3}{2},\frac{18}{11},\frac{23}{12}\right\}\subseteq (1,\;2].$$

In this example, we have ${ƛ}_{\overset{˙}{ı}}=1$, $\left(\overset{˙}{ı}=1,2,3\right)$, ${\gamma_{2}}_{1}=\frac{1}{3}$, ${\gamma_{2}}_{2}=\frac{3}{4}$, ${\gamma_{2}}_{3}=\frac{1}{2}$, $A_{1}=A_{2}=2$, ${\zeta_{1}}_{1}=\frac{1}{5}\in (0,T)$, ${\zeta_{1}}_{2}=\frac{1}{3}\in (0,T)$ and $\Delta (\vartheta ,\xi )=\frac{1}{32\pi (\vartheta e^{\vartheta^{2}}+1)}\xi (\vartheta )$,

$${\overset{ˆ}{\Delta }}_{1}(\lambda ,\xi )=\frac{\xi (\vartheta )}{e^{\vartheta }(15+\pi )},\;{\overset{ˆ}{\Delta }}_{2}(s,\xi )=\frac{\sin(\xi (\vartheta ))}{20}+\ln(\vartheta +2),{\overset{ˆ}{\Delta }}_{3}(s,\xi )=\frac{\xi (s)}{15(e^{2\vartheta }\ln(\vartheta +1))}+\tan(\vartheta +1).$$

Also for $\xi ,\overset{ˆ}{\xi }\in R$ and ϑ∈[0,T], we have

$$|\Delta (\vartheta ,\xi )-\Delta (\vartheta ,\overset{ˆ}{\xi })|=|\frac{\xi (\vartheta )}{32\pi (\vartheta e^{\vartheta^{2}}+1)}-\frac{\overset{ˆ}{\xi }(\vartheta )}{32\pi (\vartheta e^{\vartheta^{2}}+1)}|\leq \frac{1}{32\pi }|\xi -\overset{ˆ}{\xi }|,$$

and 2

$$|{\overset{ˆ}{\Delta }}_{1}(\vartheta ,\xi )-{\overset{ˆ}{\Delta }}_{1}(\vartheta ,\overset{ˆ}{\xi })|=|\frac{\xi (\vartheta )}{e^{\vartheta }(15+\pi )}-\frac{\xi (\vartheta )}{e^{\vartheta }(15+\pi )}|\leq \frac{1}{15+\pi }|\xi -\overset{ˆ}{\xi }|,|{\overset{ˆ}{\Delta }}_{2}(\vartheta ,\xi )-{\overset{ˆ}{\Delta }}_{2}(\vartheta ,\overset{ˆ}{\xi })|=|\frac{\sin(\xi (\vartheta ))}{20}+\ln(\vartheta +2)-\frac{\sin(\overset{ˆ}{\xi }(\vartheta ))}{20}-\ln(\vartheta +2)|\leq \frac{1}{20}|\xi -\overset{ˆ}{\xi }|,|{\overset{ˆ}{\Delta }}_{3}(\vartheta ,\xi )-{\overset{ˆ}{\Delta }}_{3}(\vartheta ,\overset{ˆ}{\xi })|=|\frac{\xi (s)}{15(e^{2\vartheta }\ln(\vartheta +1))}+\tan(\vartheta +1)-\frac{\xi (s)}{15(e^{2\vartheta }\ln(\vartheta +1))}-\tan(\vartheta +1)|\leq \frac{1}{15}|\xi -\overset{ˆ}{\xi }|.$$

Hence, $\psi_{1}^{⁎}=\frac{1}{32\pi }$, $\psi_{2}^{⁎}=\frac{1}{15+\pi }$, $\psi_{3}^{⁎}=\frac{1}{20}$, $\psi_{4}^{⁎}=\frac{1}{15}$, $\psi^{⁎}=\max\{\psi_{\overset{˙}{ı}}^{⁎}\;:\;\overset{˙}{ı}=1,\ldots ,4\}=0,06666$, and by using (3.5), we obtain

$$ℵ≃\left\{ \begin{array}{cc} 12.6877,\; & \gamma_{1}=\frac{3}{2},\\ 10.2871,\; & \gamma_{1}=\frac{18}{11},\\ 7.4404,\; & \gamma_{1}=\frac{23}{12}. \end{array}\right.$$

Therefore, we have

$$\psi^{⁎}ℵ≃\left\{\begin{array}{cc} 0.8458,\; & \gamma_{1}=\frac{3}{2},\\ 0.6858,\; & \gamma_{1}=\frac{18}{11},\\ 0.4960,\; & \gamma_{1}=\frac{23}{12}. \end{array}\right\}<1.$$

**Figure 1:**
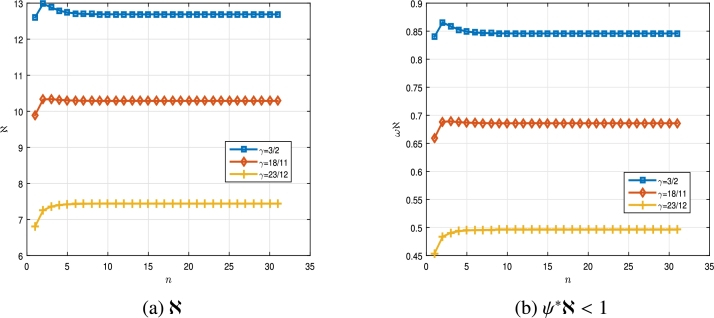
2D plot of ℵ and *ψ*^⁎^ℵ for multi-point BVP (4.1) in Example 4.1.

In Figs. 1a and 1b, the results of ℵ and $\psi^{⁎}ℵ<1$ are plotted for the multi-point BVP (4.1) when $\gamma_{1}\in \{\frac{3}{2},\frac{18}{11},\frac{23}{12}\}$. The results shown in Table 1 are obtained for the multi-point BVP (4.1) based on the definitions stated in the second section. One can use the Algorithm 3 for reproducing these obtained numerical results. Hence, all the hypotheses of Theorem 3.2 are satisfied. Thus, by the conclusion of Theorem 3.2, multi-point BVP (4.1) has a unique solution. As Figs. 1a and 1b show, as $\gamma_{1}$ increases close to 2, ℵ and $\psi^{⁎}ℵ$ parameters decrease, but the condition $\psi^{⁎}ℵ<1$ is still valid. Therefore, the nonlinear multi-point BVP of FqDEs with two RL *q*-integrals of fractional order (1.1), confirm the correctness of our results in this case.

**Table 1:** Numerical results for Γ_*q*_, ℵ and *ψ*^⁎^ℵ in Example 4.1.

*n*	$\gamma_{1}=\frac{3}{2}$			$\gamma_{1}=\frac{18}{11}$			$\gamma_{1}=\frac{23}{12}$		
	Γ_*q*_(*γ*_1_ + 1)	ℵ	*ψ*^⁎^ℵ < 1	Γ_*q*_(*γ*_1_ + 1)	ℵ	*ψ*^⁎^ℵ < 1	Γ_*q*_(*γ*_1_ + 1)	ℵ	*ψ*^⁎^ℵ < 1
1	2.8284	12.6040	0.8403	3.1088	9.8897	0.6593	3.7755	6.8018	0.4535
2	2.3270	12.9814	0.8654	2.5355	10.3276	0.6885	3.0324	7.2511	0.4834
3	2.0684	12.8812	0.8587	2.2476	10.3373	0.6892	2.6768	7.3579	0.4905
4	1.9445	12.7906	0.8527	2.1116	10.3155	0.6877	2.5125	7.3997	0.4933
5	1.8847	12.7399	0.8493	2.0463	10.3015	0.6868	2.4343	7.4199	0.4947
6	1.8554	12.7138	0.8476	2.0144	10.2943	0.6863	2.3963	7.4301	0.4953
7	1.8409	12.7007	0.8467	1.9987	10.2907	0.6860	2.3776	7.4352	0.4957
8	1.8338	12.6942	0.8463	1.9909	10.2889	0.6859	2.3683	7.4378	0.4959
9	1.8302	12.6910	0.8461	1.9870	10.2880	0.6859	2.3637	7.4391	0.4959
10	1.8284	12.6893	0.8460	1.9850	10.2875	$\underline{0.6858}$	2.3614	7.4398	$\underline{0.4960}$
11	1.8275	12.6885	0.8459	1.9841	10.2873	0.6858	2.3602	7.4401	0.4960
12	1.8270	12.6881	0.8459	1.9836	10.2872	0.6858	2.3597	7.4402	0.4960
13	1.8268	12.6879	0.8459	1.9833	10.2872	0.6858	2.3594	7.4403	0.4960
14	1.8267	12.6878	0.8459	1.9832	$\underline{10.2871}$	0.6858	2.3592	$\underline{7.4404}$	0.4960
15	1.8267	12.6878	0.8459	1.9832	10.2871	0.6858	2.3592	7.4404	0.4960
16	1.8266	$\underline{12.6877}$	$\underline{0.8458}$	1.9831	10.2871	0.6858	2.3591	7.4404	0.4960
17	1.8266	12.6877	0.8458	1.9831	10.2871	0.6858	2.3591	7.4404	0.4960
⋮	⋮	⋮	⋮	⋮	⋮	⋮	⋮	⋮	⋮

In the next example, the changes of the variable *q* have been taken into account and we consider the derivative order of $\gamma_{1}$ to be constant.

Example 4.2 Let us consider the following multi-point BVP,

(4.2) {Dq32Cξ(ϑ)=Δ(ϑ,ξ(ϑ))+∑ı˙=1mƛı˙Iqγ2ı˙Δˆı˙(ϑ,ξ(ϑ)),m=3,Iq12ξ(0)=0,Dq12ξ(1)=∑ȷ˙=1lAȷ˙Iq12ξ(ζ1ȷ˙),l=2,

for ϑ∈[0,T]=[0,1], with T=1, $\gamma_{1}=\frac{3}{2}\in (1,2]$ and three values of

$$q\in \left\{\frac{2}{11},\frac{4}{11},\frac{6}{11}\right\}\subseteq (0,\;1).$$

We take ${ƛ}_{1}=1.5$, ${ƛ}_{2}=2.3$, ${ƛ}_{3}=1.7$, ${\gamma_{2}}_{1}=\frac{1}{8}$, ${\gamma_{2}}_{2}=\frac{7}{9}$, ${\gamma_{2}}_{3}=\frac{5}{8}$, $A_{1}=2.2$, $A_{2}=3.1$, ${\zeta_{1}}_{1}=\frac{1}{7}\in (0,T)$, ${\zeta_{1}}_{2}=\frac{2}{5}\in (0,T)$ and Δ(ϑ,ξ), ${\overset{ˆ}{\Delta }}_{1}(s,\xi )$, ${\overset{ˆ}{\Delta }}_{2}(s,\xi )$, ${\overset{ˆ}{\Delta }}_{3}(s,\xi )$, be the same functions as the previous example. Thus, for $\xi ,\overset{ˆ}{\xi }\in R$ and ϑ∈[0,T], we have $\left|\Delta (\vartheta ,\xi )-\Delta (\vartheta ,\overset{ˆ}{\xi })|\leq \psi_{1}^{⁎}|\xi -\overset{ˆ}{\xi }\right|$ with $\psi_{1}^{⁎}=\frac{1}{32\pi }$, and

$$|{\overset{ˆ}{\Delta }}_{1}(\vartheta ,\xi )-{\overset{ˆ}{\Delta }}_{1}(\vartheta ,\overset{ˆ}{\xi })|\leq \psi_{2}^{⁎}|\xi -\overset{ˆ}{\xi }|,|{\overset{ˆ}{\Delta }}_{2}(\vartheta ,\xi )-{\overset{ˆ}{\Delta }}_{2}(\vartheta ,\overset{ˆ}{\xi })|\leq \psi_{3}^{⁎}|\xi -\overset{ˆ}{\xi }|,|{\overset{ˆ}{\Delta }}_{3}(\vartheta ,\xi )-{\overset{ˆ}{\Delta }}_{3}(\vartheta ,\overset{ˆ}{\xi })|\leq \psi_{4}^{⁎}|\xi -\overset{ˆ}{\xi }|,$$

where $\psi_{2}^{⁎}=\frac{1}{15+\pi }$, $\psi_{3}^{⁎}=\frac{1}{20}$, $\psi_{4}^{⁎}=\frac{1}{15}$, and $\psi^{⁎}≃0,06666$. Now, by employing (3.5), we obtain

$$ℵ≃\left\{ \begin{array}{cc} 11.2585,\; & q=\frac{2}{11},\\ 11.0574,\; & q=\frac{4}{11},\\ 10.5466,\; & q=\frac{6}{11}, \end{array}\right.\;\psi^{⁎}ℵ≃\left\{\begin{array}{cc} 0.7505,\; & q=\frac{2}{11},\\ 0.7371,\; & q=\frac{4}{11},\\ 0.7031,\; & q=\frac{6}{11}, \end{array}\right\}<1.$$

**Figure 2:**
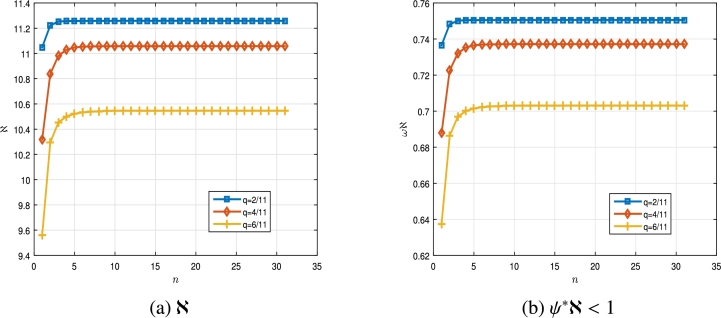
2D plot of ℵ and *ψ*^⁎^ℵ for multi-point BVP (4.2) in Example 4.2.

In Figs. 2a and 2b, the results of ℵ and $\psi^{⁎}ℵ<1$ are plotted for the multi-point BVP (4.2) when $q\in \{\frac{2}{11},\frac{4}{11},\frac{6}{11}\}$. The results shown in Table 2 are obtained for the multi-point BVP (4.2) based on the definitions stated in the second section. Hence, all the hypotheses of Theorem 3.2 are satisfied. Thus, by the conclusion of Theorem 3.2, multi-point BVP (4.2) has a unique solution. As we have considered the value of *q* between zero and 1 from the beginning, Table 2 shows that as *q* increases and approaches 1, the ℵ and $\psi^{⁎}ℵ$ parameters decrease. To reproduce these obtained results see the Algorithm 4. Therefore, the nonlinear multi-point BVP of FqDEs with two RL *q*-integrals of fractional order (1.1), confirm the correctness of our results in this case too.

**Table 2:** Numerical results for Γ_*q*_, ℵ and *ψ*^⁎^ℵ in Example 4.2.

*n*	$q=\frac{2}{11}$			$q=\frac{4}{11}$			$q=\frac{6}{11}$		
	Γ_*q*_(*γ*_1_ + 1)	ℵ	*ψ*^⁎^ℵ < 1	Γ_*q*_(*γ*_1_ + 1)	ℵ	*ψ*^⁎^ℵ < 1	Γ_*q*_(*γ*_1_ + 1)	ℵ	*ψ*^⁎^ℵ < 1
1	1.3512	11.0490	0.7366	1.9699	10.3178	0.6879	3.2631	9.5601	0.6373
2	1.3099	11.2228	0.7482	1.7605	10.8378	0.7225	2.6044	10.2947	0.6863
3	1.3021	11.2521	0.7501	1.6797	10.9809	0.7321	2.2408	10.4528	0.6969
4	1.3007	11.2574	0.7505	1.6506	11.0298	0.7353	2.0543	10.5011	0.7001
5	1.3004	11.2583	$\underline{0.7506}$	1.6402	11.0474	0.7365	1.9576	10.5221	0.7015
6	1.3004	$\underline{11.2585}$	0.7506	1.6364	11.0538	0.7369	1.9066	10.5331	0.7022
7	1.3003	11.2585	0.7506	1.6350	11.0561	0.7371	1.8793	10.5391	0.7026
8	1.3003	11.2585	0.7506	1.6345	11.0569	0.7371	1.8646	10.5425	0.7028
9	1.3003	11.2585	0.7506	1.6343	11.0573	$\underline{0.7372}$	1.8567	10.5444	0.7030
10	1.3003	11.2585	0.7506	1.6342	$\underline{11.0574}$	0.7372	1.8523	10.5454	0.7030
11	1.3003	11.2585	0.7506	1.6342	11.0574	0.7372	1.8500	10.5460	$\underline{0.7031}$
12	1.3003	11.2585	0.7506	1.6342	11.0574	0.7372	1.8487	10.5463	0.7031
13	1.3003	11.2585	0.7506	1.6342	11.0574	0.7372	1.8480	10.5464	0.7031
14	1.3003	11.2585	0.7506	1.6342	11.0574	0.7372	1.8476	10.5465	0.7031
15	1.3003	11.2585	0.7506	1.6342	11.0574	0.7372	1.8474	$\underline{10.5466}$	0.7031
16	1.3003	11.2585	0.7506	1.6342	11.0574	0.7372	1.8473	10.5466	0.7031
17	1.3003	11.2585	0.7506	1.6342	11.0574	0.7372	1.8472	10.5466	0.7031
⋮	⋮	⋮	⋮	⋮	⋮	⋮	⋮	⋮	⋮

In the next Example 4.3, we check the correctness of the results of Theorem 3.4. For this purpose, we consider several different values for *q*.

Example 4.3 As a third illustrative example, let us take multi-point BVP,

(4.3) {Dq43ξ(ϑ)=Δ(ϑ,ξ(ϑ))+∑ı˙=1mƛı˙Iqγ2ı˙Δˆı˙(ϑ,ξ(ϑ)),ϑ∈[0,T],m=2,Iq23ξ(0)=0,Dq23ξ(1)=∑ȷ˙=1lAȷ˙Iq13ξ(ζ1ȷ˙),l=2,

for ϑ∈[0,T]=[0,1], T=1, here $\gamma_{1}=\frac{4}{3}\in (1,2]$,

$$q=\left\{\frac{6}{11},\frac{8}{11},\frac{9}{11}\right\},$$

${ƛ}_{1}=-\frac{1}{9}$, ${ƛ}_{2}=\frac{3}{14}$, ${\gamma_{2}}_{1}=\frac{1}{5}$, ${\gamma_{2}}_{2}=\frac{5}{13}$, $A_{1}=\frac{3\sqrt{3}}{2}$, $A_{2}=\frac{2}{5}$, ${\zeta_{1}}_{1}=\frac{4}{13}$, ${\zeta_{1}}_{2}=\frac{5}{19}$ and

$$\Delta (\vartheta ,\xi )=\frac{e^{\vartheta }}{\left(1+e^{\vartheta })(15|\xi |+\vartheta +7\right)}\left(\xi^{2}(\vartheta )-\frac{1}{5}\right),{\overset{ˆ}{\Delta }}_{1}(\vartheta ,\xi )=\frac{\xi (\vartheta )}{7\pi +\vartheta^{2}}+\frac{3}{5\pi +10\vartheta +3}\left(1+e^{\vartheta +1}\right),{\overset{ˆ}{\Delta }}_{2}(\vartheta ,\xi )=\frac{\xi^{2}(\vartheta )}{\vartheta +1+20\pi |\xi |}\sqrt{1-\frac{\vartheta^{3}}{2}}+\frac{1}{10\pi +5\vartheta }\left(1+\tanh\left(\pi \vartheta +\frac{1}{2}\right)\right).$$

Then, thanks to Eqs. (3.10) and (3.11), we can find that

∇1=T43(1−q)43∑k=0∞ql(q43;q)k(q;q)k+Γq(73)T|[43]qT13−E|(∑ȷ˙=1k|Aȷ˙|ζ1ȷ˙53(1−q)53∑k=0∞qk(q53;q)k(q;q)k)+T23(1−q)23∑k=0∞qk(q23;q)k(q;q)k≃{2.2308,q=211,1.9751,q=411,1.5606,q=611,∇2=T2315(1−q)2315∑k=0∞qk(q2315;q)k(q;q)k+Γq(73)T|[43]qT13−E|(∑j=1l|Aȷ˙|ζ1ȷ˙2815(1−q)2815∑k=0∞qk(q2815;q)k(q;q)k)+T1315(1−q)1315∑k=0∞qk(q1415;q)k(q;q)k≃{2.0269,q=211,1.7884,q=411,1.4728,q=611,∇3=T6739(1−q)6739∑k=0∞qk(q6739;q)k(q;q)k+Γq(73)T|[73]qT13−E|(∑j=12|Aȷ˙|ζ1ȷ˙8039(1−q)8039∑k=0∞qk(q8039;q)k(q;q)k)+T4139(1−q)4139∑k=0∞qk(q4139;q)k(q;q)k≃{1.7669,q=211,1.5792,q=411,1.3999,q=611.

Clearly,

$$|\Delta (\vartheta ,\xi )|=\left|\frac{e^{\vartheta }}{\left(1+e^{\vartheta })(15|\xi |+\vartheta +7\right)}\left(\xi^{2}(\vartheta )-\frac{1}{5}\right)\right|\leq \left|\frac{e^{\vartheta }}{1+e^{\vartheta }}\right|\;\left|\frac{\xi^{2}(\vartheta )}{\left(15|\xi |+\vartheta +7\right)}\right|\leq \frac{e^{\vartheta }}{1+e^{\vartheta }}\;\left|\frac{\xi^{2}(\vartheta )}{15|\xi |}\right|\leq \overset{ˆ}{b}(\vartheta )\;\frac{\left|\xi \right|}{15},$$

and

$$|{\overset{ˆ}{\Delta }}_{1}(\vartheta ,\xi )|=\left|\frac{\xi (\vartheta )}{7\pi +\vartheta^{2}}+\frac{3(1+e^{\vartheta +1})}{5\pi +10\vartheta +3}\right|\leq {\overset{ˆ}{b}}_{1}(\vartheta )(5|\xi |+21),|{\overset{ˆ}{\Delta }}_{2}(\vartheta ,\xi )|=\left|\frac{\xi^{2}(\vartheta )}{\vartheta +1+20\pi |\xi |}\sqrt{1-\frac{\vartheta^{3}}{2}}+\frac{1}{10\pi +5\vartheta }\left(1+\tanh\left(\pi \vartheta +\frac{1}{2}\right)\right)\right|\leq {\overset{ˆ}{b}}_{2}(\vartheta )\left(\left|\xi \right|+2\right),$$

such that

$$\overset{ˆ}{b}(\vartheta )=\frac{e^{\vartheta }}{5(1+e^{\vartheta })},\;{\overset{ˆ}{b}}_{1}(\vartheta )=\frac{1+e^{\vartheta +1}}{35\pi },\;{\overset{ˆ}{b}}_{2}(\vartheta )=\frac{1}{20\pi }\left(1+\tanh\left(\pi \vartheta +\frac{1}{2}\right)\right),$$

and $\Delta \left(\left|\xi \right|\right)=\frac{\left|\xi \right|}{15}$, $\Delta_{1}\left(\left|\xi \right|\right)=5\left|\xi \right|+21$, $\Delta_{2}(|\xi |)=|\xi |+2$. Hence,

$$\|\overset{ˆ}{b}(\vartheta )\|=\frac{e}{10},\;\|{\overset{ˆ}{b}}_{1}(\vartheta )\|=\frac{1}{35\pi }(1+e^{2}),\;\|{\overset{ˆ}{b}}_{2}(\vartheta )\|=\frac{1}{20\pi }\left(1+\tanh\left(\pi +0.5\right)\right),$$

and eventually, by applying accurate calculation, from inequality (3.9), we can show that

(4.4) $$\|\overset{ˆ}{b}\|\frac{N}{15}\nabla_{1}+\left|{ƛ}_{1}\right|\;\|{\overset{ˆ}{b}}_{1}\|(5N+21)\nabla_{2}+\left|{ƛ}_{2}\right|\;\|{\overset{ˆ}{b}}_{2}\|(N+2)\nabla_{3}≃\left\{\begin{array}{cc} 0.4740,\; & q=\frac{2}{11},\\ 0.4304,\; & q=\frac{4}{11},\\ 0.3616,\; & q=\frac{6}{11}, \end{array}\right\}<\frac{3}{4},$$

whenever $N\geq \frac{3}{4}$. The curves drawn in Fig. 4, which are all lower than the line $y=\frac{3}{4}$, show the accuracy of condition (H4) in Theorem 3.4. This implies that, according to hypothesis (H4) in Theorem 3.4, the multi-point BVP (4.3) has at least one solution on [0,T]. The numerical results in Table 4 as well as curves 3a, 3b and 3c clearly show that not only the conditions of Theorem 3.4 are maintained, but also that as the value of *q* increases towards the number 1, the values of $\nabla_{i}$, i=1,2,3 decrease. Algorithm 5 can be used well for reproducing the numerical data in Table 3, Table 4.

**Figure 3:**
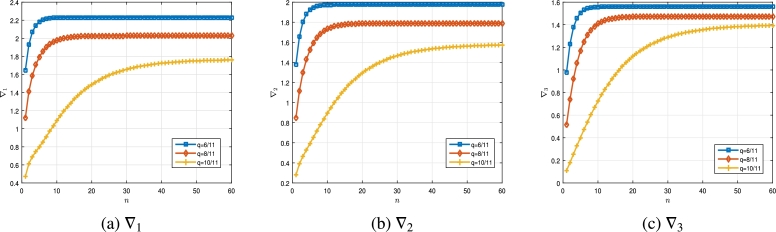
2D plot of ∇_*i*_, *i* = 1,2,3 for multi-point BVP (4.3) in Example 4.3.

**Figure 4:**
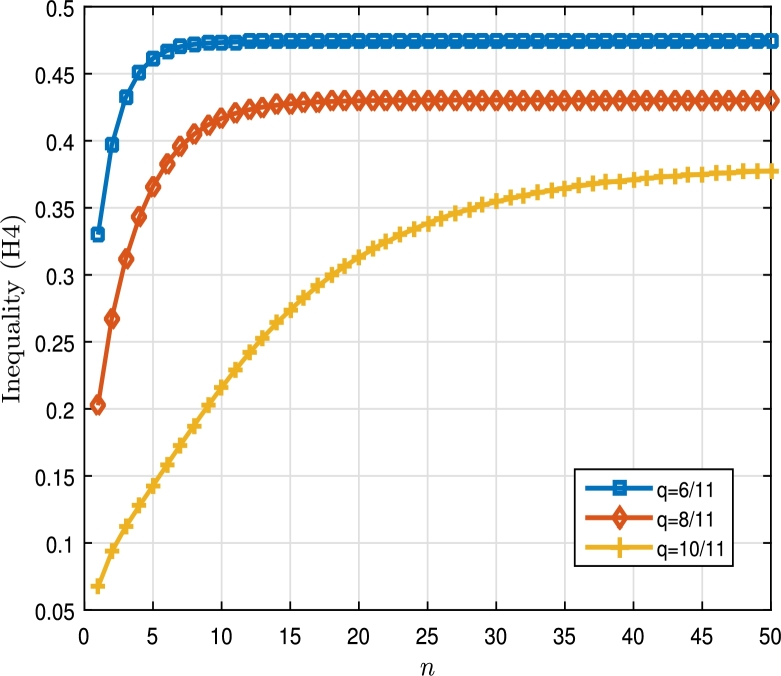
Representation of suitable *N* > 0 for inequality (4.4) for multi-point BVP (4.3) for multi-point BVP (4.3) in Example 4.3.

**Table 3:** Numerical results for ∇_1_, ∇_2_ and ∇_3_ for multi-point BVP (4.3) in Example 4.3.

*n*	$q=\frac{6}{11}$			$q=\frac{8}{11}$			$q=\frac{10}{11}$		
	∇_1_	∇_2_	∇_3_	∇_1_	∇_2_	∇_3_	∇_1_	∇_2_	∇_3_
1	1.6451	1.3792	0.9756	1.1180	0.8479	0.5132	0.4707	0.2796	0.1097
2	1.9282	1.6567	1.2300	1.4104	1.1177	0.7389	0.6138	0.3896	0.1798
3	2.0685	1.8019	1.3769	1.5872	1.2989	0.9206	0.6903	0.4660	0.2533
4	2.1427	1.8805	1.4594	1.7067	1.4285	1.0616	0.7456	0.5301	0.3275
5	2.1828	1.9234	1.5051	1.7922	1.5235	1.1688	0.7986	0.5918	0.4008
⋮	⋮	⋮	⋮	⋮	⋮	⋮	⋮	⋮	⋮
14	2.2306	1.9749	1.5604	2.0130	1.7727	1.4548	1.2862	1.0925	0.9205
15	2.2307	1.9750	1.5605	2.0168	1.7770	1.4597	1.3274	1.1336	0.9609
16	$\underline{2.2308}$	1.9750	$\underline{1.5606}$	2.0195	1.7801	1.4633	1.3654	1.1716	0.9981
17	2.2308	1.9750	1.5606	2.0216	1.7823	1.4659	1.4004	1.2067	1.0325
18	2.2308	$\underline{1.9751}$	1.5606	2.0230	1.7840	1.4678	1.4325	1.2389	1.0641
19	2.2308	1.9751	1.5606	2.0241	1.7852	1.4692	1.4619	1.2686	1.0931
20	2.2308	1.9751	1.5606	2.0249	1.7861	1.4702	1.4888	1.2957	1.1198
⋮	⋮	⋮	⋮	⋮	⋮	⋮	⋮	⋮	⋮
30	2.2308	1.9751	1.5606	2.0268	1.7883	1.4727	1.6580	1.4675	1.2890
31	2.2308	1.9751	1.5606	$\underline{2.0269}$	1.7883	$\underline{1.4728}$	1.6679	1.4776	1.2990
32	2.2308	1.9751	1.5606	2.0269	1.7883	1.4728	1.6768	1.4867	1.3080
33	2.2308	1.9751	1.5606	2.0269	$\underline{1.7884}$	1.4728	1.6850	1.4950	1.3163
34	2.2308	1.9751	1.5606	2.0269	1.7884	1.4728	1.6924	1.5026	1.3238
⋮	⋮	⋮	⋮	⋮	⋮	⋮	⋮	⋮	⋮
101	2.2308	1.9751	1.5606	2.0269	1.7884	1.4728	1.7668	1.5791	1.3998
102	2.2308	1.9751	1.5606	2.0269	1.7884	1.4728	$\underline{1.7669}$	1.5791	1.3998
103	2.2308	1.9751	1.5606	2.0269	1.7884	1.4728	1.7669	1.5791	1.3998
104	2.2308	1.9751	1.5606	2.0269	1.7884	1.4728	1.7669	1.5791	1.3998
105	2.2308	1.9751	1.5606	2.0269	1.7884	1.4728	1.7669	1.5791	1.3998
106	2.2308	1.9751	1.5606	2.0269	1.7884	1.4728	1.7669	$\underline{1.5792}$	1.3998
107	2.2308	1.9751	1.5606	2.0269	1.7884	1.4728	1.7669	1.5792	$\underline{1.3999}$
108	2.2308	1.9751	1.5606	2.0269	1.7884	1.4728	1.7669	1.5792	1.3999
109	2.2308	1.9751	1.5606	2.0269	1.7884	1.4728	1.7669	1.5792	1.3999
⋮	⋮	⋮	⋮	⋮	⋮	⋮	⋮	⋮	⋮

**Table 4:** Numerical results of hypothesis (H4) in Example 4.3.

*n*	$\|\overset{ˆ}{b}\|\frac{N}{15}\nabla_{1}$		
	$+\left|{ƛ}_{1}\right|\;\|{\overset{ˆ}{b}}_{1}\|(5N+21)\nabla_{2}+\left|{ƛ}_{2}\right|\;\|{\overset{ˆ}{b}}_{2}\|(N+2)\nabla_{3}$		
	$q=\frac{6}{11}$	$q=\frac{8}{11}$	$q=\frac{10}{11}$
1	0.3300	0.2027	0.0671
2	0.3968	0.2675	0.0935
3	0.4320	0.3114	0.1119
4	0.4510	0.3428	0.1275
5	0.4614	0.3659	0.1425
⋮	⋮	⋮	⋮
13	0.4739	0.4251	0.2532
14	0.4739	0.4266	0.2639
15	0.4739	0.4276	0.2739
16	$\underline{0.4740}$	0.4284	0.2831
17	0.4740	0.4289	0.2916
18	0.4740	0.4293	0.2994
⋮	⋮	⋮	⋮
29	0.4740	0.4303	0.3519
30	0.4740	$\underline{0.4304}$	0.3546
31	0.4740	0.4304	0.3570
32	0.4740	0.4304	0.3592
⋮	⋮	⋮	⋮
96	0.4740	0.4304	0.3815
97	0.4740	0.4304	$\underline{0.3816}$
98	0.4740	0.4304	0.3816
99	0.4740	0.4304	0.3816
⋮	⋮	⋮	⋮

## 5 Conclusion

We investigate the existence and uniqueness of solutions for a multi-point BVP involving nonlinear FDEs with two distinct fractional derivatives. Our goal is to determine whether a unique solution exists and whether it can be effectively identified. Using various FPTs, such as Banach and Leray-Schauder degree, we establish the existence of solutions. To demonstrate the validity of our findings, we provide some illustrative examples that support and confirm our results. Finally, we explore potential approaches for solving more complex mathematical problems. The boundary conditions considered are general, encompassing a range of simpler forms frequently encountered in FDEs, and our work can be extended to the framework of (p,q)-calculus for further study.

## Funding

The publication of this research was supported by the University of Oradea.

## Authors' contributions

All authors are equally contributed, read and approved the final manuscript.

## CRediT authorship contribution statement

**Isra Al-Shbeil:** Writing – original draft, Supervision, Methodology, Formal analysis. **Houari Bouzid:** Validation, Methodology, Formal analysis. **Benali Abdelkader:** Writing – review & editing, Project administration, Investigation. **Alina Alp Lupas:** Writing – review & editing, Software. **Mohammad Esmael Samei:** Validation, Resources, Methodology. **Reem K. Alhefthi:** Writing – review & editing, Validation, Funding acquisition, Data curation.

## Declaration of Competing Interest

The authors declare that they have no known competing financial interests or personal relationships that could have appeared to influence the work reported in this paper.

## Data Availability

Data sharing not applicable to this article as no datasets were generated or analyzed during the current study.
